# Expression patterns and genetic variation of the ovine skeletal muscle transcriptome of sheep from five Spanish meat breeds

**DOI:** 10.1038/s41598-018-28760-9

**Published:** 2018-07-11

**Authors:** A. Noce, T. F. Cardoso, A. Manunza, A. Martínez, A. Cánovas, A. Pons, L. A. Bermejo, V. Landi, A. Sànchez, J. Jordana, J. V. Delgado, S. Adán, J. Capote, O. Vidal, M. Pazzola, G. M. Vacca, J. Casellas, M. Amills

**Affiliations:** 1 Department of Animal Genetics, Centre for Research in Agricultural Genomics (CRAG) CSIC-IRTA-UAB-UB, Campus de la Universitat Autònoma de Barcelona, Bellaterra, 08193 Barcelona Spain; 2 Dipartimento di Medicina Veterinaria, Università degli Studi di Sassari, via Vienna, 2, 07100 Sassari, Italy; 3 CAPES Foundation, Ministry of Education of Brazil, Brasilia - D. F., Zip Code 70.040-020, Brasilia, Brazil; 4 Departamento de Genética, Universidad de Córdoba, Córdoba, 14071 Spain; 5 Centre for Genetic Improvement of Livestock, Department of Animal Biosciences, University of Guelph, Guelph, N1G 2W1 Ontario Canada; 6 Unitat de Races Autòctones, Servei de Millora Agrària i Pesquera (SEMILLA), Son Ferriol, 07198 Spain; 7 Departamento de Ingeniería, Producción y Economía Agrarias, Universidad de La Laguna, 38071 La Laguna, Tenerife Spain; 8 Departament de Ciència Animal i dels Aliments, Universitat Autònoma de Barcelona, Bellaterra, 08193 Spain; 9 Federación de Razas Autóctonas de Galicia (BOAGA), Pazo de Fontefiz, 32152 Coles, Ourense Spain; 10 Instituto Canario de Investigaciones Agrarias, La Laguna, 38108 Tenerife Spain; 11 Departament de Biologia, Universitat de Girona, Girona, 17071 Spain

## Abstract

The goal of the current study is to analyse the gene expression profile of the ovine skeletal muscle as well as to characterize the genetic variation of transcripts expressed in such tissue. This aim has been achieved by sequencing the *longissimus dorsi* transcriptomes of 50 sheep distributed in five pools representing the Canaria de Pelo, Roja Mallorquina, Gallega, Xisqueta and Ripollesa Spanish autochthonous breeds. Approximately, 363 million reads per pool have been produced and 71.9–82.9% have been successfully mapped to the ovine genome in a paired-end mode (2 × 75 bp). The 200 most expressed muscle transcripts (≈1% of the total transcript count) account for 51% (Canaria de Pelo) to 67% (Gallega) of the total ovine skeletal muscle mRNA expression. These highly expressed genes play key roles in pathways related with striated muscle contraction, gluconeogenesis, glycolysis, citric acid cycle and respiratory electron transport. RNA-Sequencing of muscle transcripts has also revealed that ~72% of the SNPs detected with this approach are shared by at least two pools, and 10% of them segregate in the five pools under analysis. Most of the substitutions detected by RNA-Seq are synonymous or missense and only a minority are predicted to have consequences on protein function.

## Introduction

The development of next generation sequencing techniques has made possible to characterize in depth the ovine transcriptome across multiple tissues. Recently, a high resolution atlas of gene expression in sheep was generated by combining data from 441 RNA-Seq libraries representing all major organ systems^1^. This study highlighted the existence of at least 25,350 expressed genes, of which 19,921 encode proteins^1^. Moreover, hundreds of genes were functionally annotated based on their co-expression patterns with loci with a well-known function^1^. Massive sequencing of transcripts expressed in specific ovine tissues, such as skeletal muscle and the mammary gland^2–4^, has also been carried out to better understand the physiology of these organs and its impact on production performance. RNA-Seq has also been used to generate large collections of single nucleotide polymorphisms (SNPs) mapping to transcripts expressed in certain tissues^2,3^, though SNP calling from sequencing data can be challenging due to the intrinsic complexity of the transcriptome^5,6^. In a recent study, Suárez-Vega *et al*.^7^ identified, through RNA-Seq of the milk somatic cell transcriptome, 197,948 SNPs in eight Churra and Assaf ewes. Zhang *et al*.^2^ detected 40,481 and 38,851 potential SNPs in transcripts expressed in the skeletal muscle of Small-Tailed Han and Dorper sheep, respectively. Characterizing the levels of expression and variation of genes transcribed in the ovine skeletal muscle may shed light on the physiological mechanisms that regulate muscle growth and development, two key aspects determining the economic output of meat sheep producers.

The current study aimed to analyse the gene expression profile of the ovine skeletal muscle by sequencing RNA extracted from *longissimus dorsi* samples of 50 individuals distributed in pools representing five meat sheep breeds from Spain *i*.*e*. Canaria de Pelo, Roja Mallorquina, Gallega, Xisqueta and Ripollesa (Supplementary Table S1). Our goal was to identify the genes making a major contribution to the muscle transcriptome and to determine their biological functions with the aim of providing insights into the molecular basis of meat traits. Moreover, we were interested in characterizing the SNP variation of transcripts expressed in the ovine skeletal muscle and to investigate if such variation is shared across breeds with different origins and demographic histories.

## Methods

### Ethics statement

Biological samples were extracted from the carcasses of sheep slaughtered in a commercial abattoir. The killing of these animals was exclusively due to the fact that they were at the end of their productive cycle and followed the Spanish legislation expressed in the Royal Decree 37/2014 which regulates animal welfare during slaughtering procedures. Given that this is a standard and routine farming procedure not directly related with our research, no permission from the Ethics Committee of the UAB was required.

### RNA isolation and sequencing

Samples from the *longissimus dorsi* muscle were retrieved from Canaria de Pelo, Roja Mallorquina, Xisqueta, Ripollesa and Gallega sheep (10 individuals per population) in a commercial abattoir. Muscle samples were submerged into RNAlater (Ambion, Austin, TX) and stored at −20 °C until use. Total RNA was extracted using the RiboPure RNA Purification kit (Ambion) following the recommendations of the manufacturer. Total RNA concentration was estimated with a Nano-Drop ND-1000 spectrophotometer (NanoDrop products; Wilmington, USA). The quality of the RNA extractions was evaluated in an Agilent Bioanalyzer 2100 equipment (Agilent Technologies, Inc., Santa Clara CA, USA). All samples showed RNA Integrity Numbers (RIN) values above 7. Subsequently, five RNA pools were prepared by mixing equal RNA amounts from each one of the 10 individuals representing a given breed. Sequencing libraries (five cDNA pools) were made using the TruSeq RNA Sample Preparation kit (Illumina, San Diego, CA) and following the protocols recommended by the manufacturer. The TruSeq RNA Kit captures the coding transcriptome (without strand information) by using oligo-dT beads complementary to poly-A tails. RNA paired-end sequencing (2 × 75 bp) was carried out in a HiSeq 2000 Sequencing System (Illumina, San Diego, CA) at the Centre Nacional d’Anàlisi Genòmica (http://www.cnag.crg.eu, Barcelona, Spain).

### Bioinformatic analysis

Quality control of sequence reads was carried out with the CLC Genomics Workbench 8.0 (https://www.qiagenbioinformatics.com), by using the NGS quality control tool, which assesses sequence quality indicators based on the FastQC-project (http://www.bioinformatics.babraham.ac.uk/projects/fastqc). Subsequently, sequences were trimmed for any remaining sequencing adapter and low quality bases by using Trimmomatic v.0.22^8^ and taking into account default parameters. Raw reads were mapped to the ovine reference genome Oar_v3.1 (https://www.ensembl.org/Ovis_aries/.) by using the Spliced Transcripts Alignment to a Reference (STAR) software^9^ with a 2-pass mapping strategy. This strategy relies on a double mapping of the reads by STAR. In the 1^st^ pass, the novel junctions are identified and included into the genome indices. In the 2^nd^ pass, all reads are mapped again by considering the original GTF file plus the novel junctions (*i*.*e*. those detected in the 1^st^ pass). To characterize muscle gene expression in each pool, we used the *FeatureCounts* tool^10^. The data inputted to *FeatureCounts* consists of one or more files of aligned reads in Binary Alignment/Map (BAM) format and a list of genomic features (GTF annotation file). *FeatureCounts* assigns reads to features by comparing the genomic location of each base in the read with the chromosomal region encompassed by each feature^10^. By combining highly efficient chromosome hashing and feature blocking techniques, *FeatureCounts* provides highly precise read counts with a low computational cost^10^. In order to normalise the raw counts, we determined the relative library sizes for each library through the function *estimateSizeFactors* of the *DESeq2* package^11^. This function is based on the median of ratios method, which performs the following steps: 1 - a pseudo-reference sample is created (that is equal to the geometric mean across all samples) for each gene; 2 - for every gene in a sample, the ratios (sample/ pseudo-reference) are calculated; 3 - the median value of all ratios for a single sample is taken as the normalization factor (size factor) for each sample and 4 - the function divides each raw count value by the normalization factor to generate normalized count values^12^. The Cytoscape software^13^ combined with the ReactomeFIViz app^14^ were used to identify which pathways are enriched in the data set of 200 genes displaying the highest levels of expression in each pool.

Variant discovery procedures followed the *GATK Best Practices workflow for SNP calling on RNA-Seq data* (https://software.broadinstitute.org/gatk/documentation/article.php?id=3891). After mapping, reads were split into exon segments and any sequences overhanging into the intronic regions were hard-clipped. Mapping qualities were reassigned by using the SplitNCigarReads GATK tool (https://software.broadinstitute.org/gatk). We used the Haplotype Caller (https://software.broadinstitute.org/gatk) tool to detect SNPs by considering the presence of ten individuals (–ploidy 20) in each pool. A Phred-scaled confidence threshold of 20 was taken into consideration. Stringent parameters were used to minimize the detection of false-positive SNPs. In this way, variants meeting any of the two following conditions were eliminated: a) with Fisher Strand values above 30.0 (the Fisher’s exact test is used to determine if there is strand bias between forward and reverse strands for the reference or alternate alleles); and b) Qual By Depth values (quality score normalized by allele depth for a variant) below 2. Indel variation was not taken into account because accurate indel calling is still difficult to implement, as reflected by the low agreement between calling algorithms^15^. We used the BEDTools software^16^ to filter out intronic and intergenic SNPs, thus retaining those that map to exons (reference genome Oar_v3.1). The potential effects and impact of exonic SNPs identified via RNA-Seq were predicted with the SnpEff software v4.3s^17^. The impact of a SNP is categorised by SnpEff as follows: HIGH, the variant is assumed to have high (disruptive) impact on the protein, probably causing protein truncation, loss of function or triggering nonsense mediated decay; MODERATE, a non-disruptive variant that might change protein effectiveness; LOW, assumed to be mostly harmless or unlikely to change protein behavior; MODIFIER, usually non-coding variants or variants affecting non-coding genes, where predictions are difficult or there is no evidence of impact (http://snpeff.sourceforge.net/SnpEff_manual.html). We considered as “previously reported variants” those described in the Single Nucleotide Polymorphism database (dbSNP)^18^.

### Data availability

The raw sequencing data used to perform RNA-Seq analysis are available in the Sequence Read Archive (SRA) BioProject No. PRJNA472958. The new polymorphisms identified in the current work can be found in Figshare^19^.

## Results and Discussion

### A few hundred genes are highly expressed in the ovine skeletal muscle

Approximately, 363 million reads per pool have been produced and 71.9–82.9% of these reads have been successfully mapped to the ovine genome in a paired-end (2 × 75 bp) mode (Table 1). Nearly 49–55% of unambiguously mapped reads correspond to exons. We have discarded from subsequent analyses intergenic (21–27%) and intronic (22–23%) reads because the functional annotation of such regions in the ovine genome is still very poor and, moreover, they could be the result of genomic DNA contamination. Based on exonic data, we have detected the existence of 14,743-15,454 expressed genes (the threshold of expression was set at >10 normalized DESeq2 counts) in the ovine skeletal muscle. The number of expressed genes detected in the current work is similar to those identified in previous reports^1,2,4^. In this way, Zhang *et al*.^2^ sequenced the muscle transcriptome of two sheep from the Dorper and Small-tailed Han breeds and detected the expression of about 13,500 known reference genes based on 50 M reads/sample (in our experiment we generated an average of 363 M reads per pool). In the sheep gene expression atlas data set^1^, 25,350 genes (19,921 protein-coding loci) with expression levels above 1 transcript per million were detected in at least one tissue from one individual^1^. Amongst the most expressed loci (Supplementary Table S2), we have found genes encoding myofibrilar proteins involved in muscle contraction (*ACTA1*, *MYLPF*, *MYH2*, *MYH7*, *TPM2* and *TTN*) as well as genes related with oxygen storage and diffusion (*MB*), mitochondrial respiration (*MT-CYB*), calcium transportation and release (*ATP2A1* and *RYR1*) and energy homeostasis (*ALDOA*, *CKM* and *GAPDH*). The pattern of expression of muscle transcripts is highly unbalanced, with a few genes contributing most of transcripts (Supplementary Table S2). For instance, the 200 most expressed transcripts (≈1% of the total transcript count) account for 51% (Canaria de Pelo), 53% (Roja Mallorquina), 62% (Xisqueta), 65% (Ripollesa) and 67% (Gallega) of the total muscle gene expression. These patterns of expression were confirmed by analysing three independent additional RNA-Seq data sets (Supplementary Table S3, Sabino *et al*.^20^). The genes identified by us as showing the highest expression levels in the *longissimus dorsi* muscle of Spanish sheep also showed the highest expression levels and similar expression rankings in Appenninica × Sarda lambs (Supplementary Table S3). This unbalanced expression profile has been also observed in human tissues^21^. For instance, 60% of the blood cell transcriptome is contributed by the three hemoglobin genes^21^. Zhang *et al*.^2^ also observed this phenomenon in the ovine skeletal muscle of Dorper and Small-Tailed Han sheep *i*.*e*. they found 12,618-12,746 genes with an expression of 0–100 RPKM, whilst only 12 genes displayed very high levels of expression (>10,000 RPKM).

**Table 1:** Output of the RNA-Sequencing of *longissimus dorsi* muscle RNA pools representing five Spanish ovine breeds (N = 10 per pool).

Pool	Total number of aligned reads	Percentage of mapped paired-end reads
Canaria de Pelo	387,913,634	75.7%
Gallega	333,455,394	80.4%
Ripollesa	305,951,754	82.9%
Roja Mallorquina	385,834,634	71.9%
Xisqueta	403,175,394	72.7%

Our results clearly indicate that the most expressed locus in the skeletal muscle tissue of the five breeds under analysis is the actin α1 (*ACTA1*) gene (Supplementary Table S2), which encodes the thin filaments of the muscle contractile apparatus^22^. Myosin heavy chain 2 (*MYH2*) and 7 (*MYH7*) and myosin light chain, phosphorylatable, fast skeletal muscle (*MYLPF*) genes are also strongly expressed (Supplementary Table S2), and their products form part of the thick filaments of muscular myofibrils, while tropomyosin 2 (*TPM2*) and titin (*TTN*) are key modulators of muscle contraction^22^. The myoglobin (*MB*) and mitochondrially encoded cytochrome B (*MT-CYB*) loci also display a high expression (Supplementary Table S2), reflecting the fundamental role of oxidative phosphorylation in the generation of the ATP needed for muscle contraction^23^. Calcium is an essential trigger of muscle contraction and by this reason the mRNA levels of the ATPase sarcoplasmic/endoplasmic reticulum Ca^2+^ transporting 1 (*ATP2A1*) gene, which translocates Ca^2+^ from the cytosol to the sarcoplasmic reticulum, and the ryanodine receptor 1 gene, that facilitates the release of Ca^2+^ into the cytosol, are expressed at high levels in the ovine skeletal muscle (Supplementary Table S2)^24^. Given the high energy expenditure of the skeletal muscle tissue, creatine kinase, M-type (*CKM*), which is involved in energy storage^25^, and aldolase, fructose-bisphosphate A (*ALDOA*) and glyceraldehyde-3-phosphate dehydrogenase (*GAPDH*), which participate in glycolysis^26^, are also strongly expressed (Supplementary Table S2). In close concordance with our data, genes mentioned above are present in the list of the 100 top expressed loci in the human skeletal muscle^21^. As shown in Supplementary Table S4, the main functions of the 200 ovine top genes listed in Supplementary Table S2 are related with muscle contraction and metabolism (gluconeogenesis, glycolysis, citric acid cycle, formation of ATP by chemiosmotic coupling and respiratory electron transport). Both groups of pathways are highly interconnected because the energy needed for the contraction of skeletal muscle fibers is supplied by ATP via anaerobic glycolysis, the phosphocreatine shuttle and oxidative phosphorylation^27^. As observed in humans^21^, nuclear genes are the main contributors to the transcriptomic output of the ovine skeletal muscle (Supplementary Table S2). In contrast, RNA-Seq experiments have demonstrated that in highly aerobic human tissues, such as kidney, 51% of the transcriptional output comes from mitochondrial genes^21^.

### About the variation of genes expressed in the ovine skeletal muscle

The amount of exonic polymorphisms detected by RNA-Seq in the Ripollesa (109,678 SNPs), Xisqueta (118,998 SNPs), Gallega (93,464 SNPs), Roja Mallorquina (101,692 SNPs) and Canaria de Pelo (104,439 SNPs) breeds does not correlate with the current census of these five populations (Table 2). The identification of SNPs from RNA-Seq data is a challenging task due to the inherent complexity of the transcriptome and also because SNP calling is greatly affected by gene expression levels and coverage *i*.*e*. SNPs in highly expressed genes are detected with a higher probability than those mapping to weakly expressed transcripts^5,6^. In consequence, the number of SNPs identified in each pool would be a very rough estimate of genetic diversity. In order to tackle this issue, we have used previously reported Ovine SNP50 BeadChip data from Ripollesa (N = 23), Xisqueta (N = 25), Gallega (N = 25), Roja Mallorquina (N = 29) and Canaria de Pelo (N = 27) sheep^28^ to estimate observed and expected heterozygosities. Individuals included in this latter data set were not sequenced in the current work, so they can be considered as an independent sample of each one of the breeds under analysis. We have observed that both diversity parameters have similar values across the five breeds despite the fact that their population sizes are very different (Table 2). Although in general population size correlates with the amount of diversity^29^, this relationship is not linear and it can be affected by the reproductive management and demographic history (bottlenecks and founder effects, admixture, etc.) of domestic breeds. For instance, the census of Holstein cattle in Canada is much larger than that of Jersey cattle, but paradoxically effective sizes are remarkably alike *i*.*e*. 115 and 55 individuals for Holstein and Jersey, respectively^30^. Moreover, the high genetic variation of the two insular ovine breeds, Canaria de Pelo and Roja Mallorquina, could be due to population admixture *e*.*g*. Roja Mallorquina sheep have a fat tail and a red coloration that is typical from certain African and Asian breeds^28^.

**Table 2:** Single nucleotide polymorphisms (SNPs) detected in five ovine Spanish breeds by RNA-Sequencing and two diversity parameters (observed and expected heterozygosities) calculated from an independent sample of individuals genotyped with the Ovine SNP50 BeadChip.

Pool	Population size	Total SNPs	Novel SNPs	H_o_	H_e_
Canaria de Pelo	3,051	104,439	31,084	0.348	0.341
Gallega	4,319	93,464	35,976	0.370	0.375
Ripollesa	36,796	109,678	42,514	0.376	0.378
Roja Mallorquina	3,912	101,692	31,832	0.375	0.365
Xisqueta	56,328	118,998	33,192	0.389	0.384

Observed (H_o_) and expected (H_e_) heterozygosities were estimated on the basis of a data set previously reported by Manunza *et al*.^28^ which included 129 individuals from the Ripollesa (N = 23), Xisqueta (N = 25), Gallega (N = 25), Roja Mallorquina (N = 29) and Canaria de Pelo (N = 27) breeds. These 129 individuals are different from the 50 sheep sequenced in the current experiment and, moreover, they are representative of the five breeds under analysis. Population sizes were retrieved from the Catalogue of Spanish livestock breeds (http://www.mapama.gob.es).

Approximately 33%, of the SNPs detected by RNA-Seq in each breed appear to be novel *i*.*e* they are not recorded in the dbSNP^18^ database. The segregation, in the five breed pools, of a substantial proportion of SNPs that are not recorded in the dbSNP^18^ database (Table 2) matches previous results obtained by Suárez-Vega *et al*.^7^. These authors characterized the variation of the milk somatic cell transcriptome from eight ewes and found that 22% of variants were not annotated in the dbSNP^18^ database (https://www.ncbi.nlm.nih.gov/projects/SNP). Similarly, sequencing of the transcriptome of muscle samples from three Limousin bulls showed that approximately 60% of the variation was unannotated^31^. Taken together, these results suggest that a substantial fraction of ovine genetic diversity remains to be identified.

**Figure 1:**
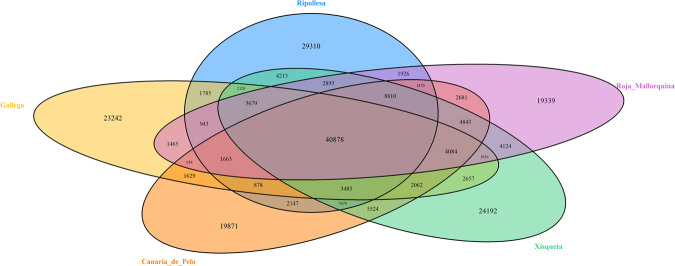
Venn-Diagram depicting the breed distribution of exonic SNPs identified in Canaria de Pelo, Roja Mallorquina, Gallega, Xisqueta and Ripollesa sheep. The total number of SNPs detected in each breed is as follows: Canaria de Pelo = 104,439 variants, Roja Mallorquina = 101,692 variants, Gallega = 93,464 variants, Xisqueta = 118,998 variants and Ripollesa = 109,678 variants.

The majority (72%) of the SNP variants identified in the current work are present in 2 or more pools, and 10% segregate in the five pools (Fig. 1). These results could be due, at least in part, to the fact that population-specific alleles have usually low frequencies^32^. When using pooled sequencing data, these rare alleles are detected with a much lower efficiency than alleles segregating at intermediate or high frequencies. This means that a robust estimate of breed-specific variation cannot be obtained from our data. However, the general picture that emerges from our results is consistent with previous studies indicating that the majority of ovine SNP variation is shared across breeds^33^. High haplotype sharing across short chromosomal distances and weak population structure observed in a worldwide sample of sheep breeds has been interpreted as evidence of extensive gene flow in ancient times^33^. However, the short time of divergence of ovine breeds (less than 10,000 YBP) and the existence of a single domestication center in the Near East might be also important causal factors explaining the extensive sharing of variants even amongst distant sheep populations.

**Figure 2:**
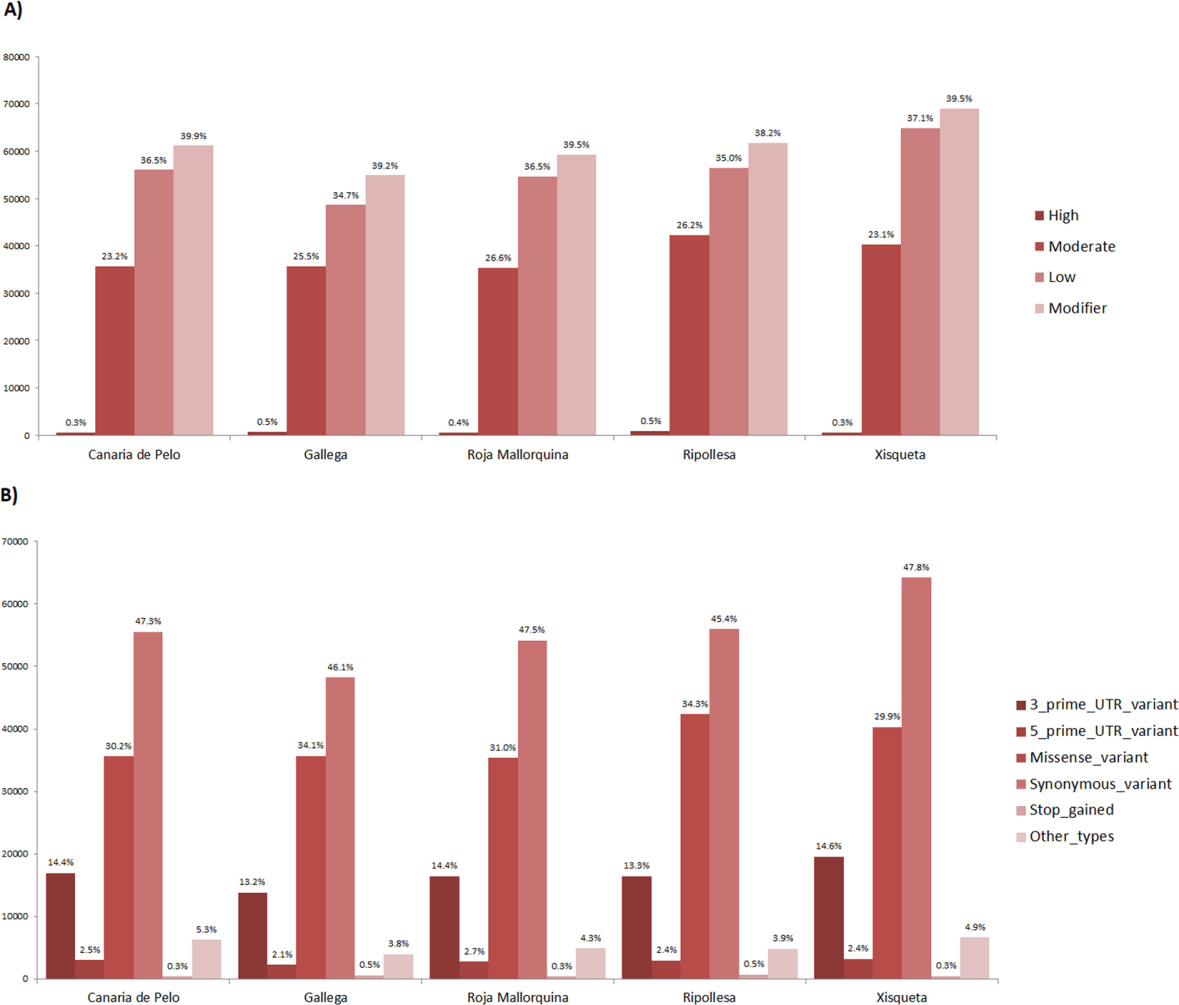
(**A**) Classification of single nucleotide polymorphisms (SNPs) identified in five ovine Spanish breeds according to their impact on gene function as predicted with the SnpEff software^17^. (**B**) Major types of SNPs detected by RNA-Seq in five Spanish ovine breeds and annotated with the SnpEff software^17^.

About the predicted functional consequences of the variation detected in the five ovine pools, high impact variants are much less frequent (less than 1% on average) than modifier (38–39%), moderate (23–26%) or low (34–37%) impact variants (Fig. 2). The vast majority of exonic SNPs are synonymous or missense or map to the 3′UTR, while those located in the 5’UTR or introducing a premature stop codon are much scarcer (Fig. 2). Nonsense variants are very scarce probably because they are purged by purifying selection (Fig. 2). Suárez-Vega *et al*.^7^ sequenced the ovine mammary gland transcriptome of Churra and Assaf sheep and found a similar pattern of variation, with ≈43,000 synonymous, ≈21,800 missense and ≈12,900 3′UTR SNPs plus 112–119 nonsense mutations.

### Detection of high impact variants in fifteen genes related with meat quality

As shown in Supplementary Table S5, we have explored the missense and nonsense variation of 15 genes related with meat quality which are listed in Supplementary Table S6. In Supplementary Table S5, we have only catalogued variants that have been independently discovered in at least two pools. The majority of missense substitutions identified in this way have been predicted to be non-damaging by SIFT (http://sift.jcvi.org/), but several of them, *i*.*e*. those mapping to the *MYF*5, *FABP4*, *PGAM2* and *PRKAG3* genes might have functional consequences (Supplementary Table S5). The variability of the *MYF5* gene has been associated with meat quality traits in pigs^34^, cattle^35^ and rabbits^36^, and a missense substitution in the porcine *PRKAG3* gene is a causal factor determining muscle glycolytic potential^37^. Allelic variation in the *PGAM2* gene has been associated with drip loss percentage in pigs^38^ and the polymorphism of *FABP4* shows associations with meat quality traits in multiple species^39^. The *FABP4* gene polymorphism is associated with meat tenderness in three Chinese native sheep breeds^40^. Moreover, we have detected twelve missense mutations in the ovine ryanodine receptor 1 (*RYR1*) gene, which has a high level of expression in the skeletal muscle, fulfills and essential role in muscle contraction and it is a strong determinant of post-mortem meat pH in pigs^41^. In humans, over 300 mutations in ryanodine receptors have been linked to severe skeletal and cardiac muscle disorders^42^. A practical application of the catalogue of mutations generated in the current work would be the genotyping of sets of selected polymorphisms in reference populations with available production records in order to investigate if they are associated with meat quality traits of economic importance for the sheep industry.

## Conclusions

In summary, our results indicate that a substantial proportion (51–67%) of the transcriptional output of the ovine skeletal muscle is contributed by a few hundred of genes (≈1% of expressed loci) which are mainly involved in muscular contraction, metabolism, calcium transport and energy homeostasis. The broad majority of SNP variants mapping to muscle transcripts are shared by at least two of the breeds under analysis, a result that could be the consequence of admixture as well as of recent divergence from a single domestication site.

## Electronic supplementary material

Supplementary Tables S1 to S6
