# Supplementary material for: Expression patterns and genetic variation of the ovine skeletal muscle transcriptome of sheep from five Spanish meat breeds
Source: Sci Rep. 2018 Jul 11;8:10486. doi: 10.1038/s41598-018-28760-9 (PMC6041298; doi:10.1038/s41598-018-28760-9)
Supplement: Supplementary file 1 — Supplementary Tables S1 to S6 [file 41598_2018_28760_MOESM1_ESM.docx]

**Expression patterns and genetic variation of the ovine skeletal muscle transcriptome of sheep from five Spanish meat breeds**

A. Noce^1,2^ ¶, T. F. Cardoso^1,3^¶, A. Manunza^1^, A. Martínez^4^, A. Cánovas^5^, A. Pons^6^, L. A. Bermejo^7^, V. Landi^4^, A. Sànchez^1,8^, J. Jordana Vidal^8^, J. V. Delgado^4^, S. Adán^9^, J. Capote^10^, O. Vidal^11^, M. Pazzola^2^, G. M. Vacca^2^, J. Casellas^8^* and M. Amills^1,8^*

^1^Department of Animal Genetics, Centre for Research in Agricultural Genomics (CRAG) CSIC-IRTA-UAB-UB, Campus de la Universitat Autònoma de Barcelona, Bellaterra 08193, Barcelona, Spain. ^2^Dipartimento di Medicina Veterinaria,Università degli Studi di Sassari, via Vienna, 2, 07100 Sassari, Italy. ^3^CAPES Foundation, Ministry of Education of Brazil, Brasilia D. F., Zip Code 70.040-020, Brazil. ^4^Departamento de Genética, Universidad de Córdoba, Córdoba 14071, Spain. ^5^Centre for Genetic Improvement of Livestock, Department of Animal Biosciences, University of Guelph, Guelph N1G 2W1, Ontario, Canada. ^6^Unitat de Races Autòctones, Servei de Millora Agrària i Pesquera (SEMILLA), Son Ferriol 07198, Spain. ^7^Departamento de Ingeniería, Producción y Economía Agrarias, Universidad de La Laguna, 38071 La Laguna, Tenerife, Spain. ^8^Departament de Ciència Animal i dels Aliments, Universitat Autònoma de Barcelona, Bellaterra 08193, Spain. ^9^Federación de Razas Autóctonas de Galicia (BOAGA), Pazo de Fontefiz, 32152 Coles. Ourense, Spain. ^10^Instituto Canario de Investigaciones Agrarias, La Laguna 38108, Tenerife, Spain. ^11^Departament de Biologia, Universitat de Girona, Girona 17071, Spain

**Supplementary Table S1. Main features and lamb growth traits of the five Spanish meat sheep breeds analysed in the current study.**

| **Breed** | **Distribution** | **Aspect** | **Growth rate (g/day)** | **Carcass weight (kg)** | **Comments** |
| --- | --- | --- | --- | --- | --- |
| Canaria de Pelo | Canary Islands | Red coloration that goes from light to dark, sometimes with a black belly. Hair instead of wool. | 150 | 8 | The breeding of Canaria de Pelo sheep is associated with the intensive production of tomatoes and bananas, whose subproducts are used to feed the livestock. |
| Roja Mallorquina | Mallorca | Intense red coloration, fat tail and polled. | 241 | 13 | Extensive production in small herds (10-300 heads), with little human intervention. Excellent adaptation to drought. |
| Gallega | Galicia | Small size and white or black coat. Males with spiral horns. | 200 | 12 | Raised in small herds, together with cows or goats. Good adaptation to mountanious environments. |
| Xisqueta | Catalonia | White coat, with black pigmentation in the ears, around the eyes and lips, and in the legs. | 260 | 12 | Summer transhumance to mountain pastures. During winter, they are fed in the pens with forage, hay and concentrate. |
| Ripollesa | Catalonia | White coat, with black or brown pigmented areas in the face and legs. | 235 | 11 | Extensive or semi-extensive breeding. Feeding based on daily grazing complemented with forage in the pen. |

**Supplementary Table S2. Genes expressed in the *longissimus dorsi* muscle of five ovine breeds**

| **XISQUETA** | | | |
| --- | --- | --- | --- |
| **Gene Ensembl ID** | **Gene name** | **Counts** | **Gene type** |
| ENSOARG00000003044 | *ACTA1* | 4548577,29 | protein_coding |
| ENSOARG00000011486 |  | 3721828,19 | protein_coding |
| ENSOARG00000009785 | *CKM* | 3069685,97 | protein_coding |
| ENSOARG00000012656 | *MYH2* | 2210832,78 | protein_coding |
| ENSOARG00000000035 | *MT-CYB* | 2091788,75 | protein_coding |
| ENSOARG00000017195 | *TTN* | 1869300,38 | protein_coding |
| ENSOARG00000004143 | *ALDOA* | 1720349,41 | protein_coding |
| ENSOARG00000006071 | *MYLPF* | 1613947,54 | protein_coding |
| ENSOARG00000007894 | *GAPDH* | 1567346,65 | protein_coding |
| ENSOARG00000019316 | *MYH7* | 1566826,44 | protein_coding |
| ENSOARG00000020797 | *TPM1* | 1201257,98 | protein_coding |
| ENSOARG00000011889 | *TPM2* | 1044548,09 | protein_coding |
| ENSOARG00000002132 | *ATP2A1* | 1024738,29 | protein_coding |
| ENSOARG00000008997 | *PYGM* | 954634,47 | protein_coding |
| ENSOARG00000000016 | *MT-CO1* | 915727,87 | protein_coding |
| ENSOARG00000018639 | *MB* | 907932,33 | protein_coding |
| ENSOARG00000005506 | *ENO3* | 807149,33 | protein_coding |
| ENSOARG00000020185 | *DES* | 743569,07 | protein_coding |
| ENSOARG00000003852 | *TNNI2* | 722078,03 | protein_coding |
| ENSOARG00000006566 | *TNNC2* | 660089,62 | protein_coding |
| ENSOARG00000013964 | *MYBPC2* | 642458,65 | protein_coding |
| ENSOARG00000005704 | *RYR1* | 623739,88 | protein_coding |
| ENSOARG00000000032 | *MT-ND5* | 600026,93 | protein_coding |
| ENSOARG00000015112 | *MYBPC1* | 551934,35 | protein_coding |
| ENSOARG00000019190 | *MYL1* | 551809,41 | protein_coding |
| ENSOARG00000003713 | *ACTN2* | 455158,97 | protein_coding |
| ENSOARG00000008659 | *NEB* | 442478,03 | protein_coding |
| ENSOARG00000017779 | *MYL2* | 442122,61 | protein_coding |
| ENSOARG00000007876 | *MYOZ1* | 433157,42 | protein_coding |
| ENSOARG00000006670 | *ACTN3* | 424782,44 | protein_coding |
| ENSOARG00000000022 | *MT-ATP6* | 401089,96 | protein_coding |
| ENSOARG00000003501 | *PDK4* | 393508,75 | protein_coding |
| ENSOARG00000018886 | *PKM* | 371935,86 | protein_coding |
| ENSOARG00000011052 |  | 338466,10 | protein_coding |
| ENSOARG00000009602 | *LDHA* | 322100,64 | protein_coding |
| ENSOARG00000008405 | *CASQ1* | 320712,35 | protein_coding |
| ENSOARG00000019300 | *PFKM* | 310512,88 | protein_coding |
| ENSOARG00000004312 | *TNNC1* | 303346,33 | protein_coding |
| ENSOARG00000013633 | *PGAM2* | 297182,50 | protein_coding |
| ENSOARG00000001823 | *TNNT1* | 285984,62 | protein_coding |
| ENSOARG00000000611 | *LDB3* | 280505,78 | protein_coding |
| ENSOARG00000003372 | *FLNC* | 268996,64 | protein_coding |
| ENSOARG00000008306 | *ATP5B* | 247009,09 | protein_coding |
| ENSOARG00000017679 |  | 246960,63 | protein_coding |
| ENSOARG00000020482 | *PDE4DIP* | 246002,07 | protein_coding |
| ENSOARG00000011575 | *TCAP* | 245566,95 | protein_coding |
| ENSOARG00000012744 | *NRAP* | 242528,65 | protein_coding |
| ENSOARG00000005466 | *TRIM63* | 241768,27 | protein_coding |
| ENSOARG00000005438 | *OBSCN* | 229397,51 | protein_coding |
| ENSOARG00000000033 | *MT-ND6* | 223402,77 | protein_coding |
| ENSOARG00000011424 | *FHL1* | 221321,95 | protein_coding |
| ENSOARG00000016226 | *ATP2A2* | 218740,31 | protein_coding |
| ENSOARG00000003388 | *KLHL41* | 201574,62 | protein_coding |
| ENSOARG00000007666 | *SLC25A4* | 200189,56 | protein_coding |
| ENSOARG00000007924 | *PDLIM3* | 196071,00 | protein_coding |
| ENSOARG00000017434 | *CMYA5* | 195263,23 | protein_coding |
| ENSOARG00000020658 | *TXNIP* | 194675,17 | protein_coding |
| ENSOARG00000020919 | *YBX3* | 184993,76 | protein_coding |
| ENSOARG00000010213 | *MYOM1* | 184924,83 | protein_coding |
| ENSOARG00000020020 | *TUBA4A* | 174637,04 | protein_coding |
| ENSOARG00000018001 | *IGFN1* | 172121,10 | protein_coding |
| ENSOARG00000018307 | *ACO2* | 163055,75 | protein_coding |
| ENSOARG00000013116 | *CA3* | 161740,70 | protein_coding |
| ENSOARG00000000037 |  | 161238,80 | Mt_tRNA |
| ENSOARG00000005748 | *MYOM2* | 159554,33 | protein_coding |
| ENSOARG00000014988 | *AHNAK* | 159450,93 | protein_coding |
| ENSOARG00000011109 | *EEF2* | 157492,89 | protein_coding |
| ENSOARG00000014048 | *CCNG1* | 143367,66 | protein_coding |
| ENSOARG00000001845 | *TPM3* | 140066,56 | protein_coding |
| ENSOARG00000018666 | *RPLP1* | 139954,55 | protein_coding |
| ENSOARG00000016562 | *GNAS* | 138255,00 | protein_coding |
| ENSOARG00000009826 | *PGM1* | 133269,43 | protein_coding |
| ENSOARG00000026131 |  | 132930,17 | lincRNA |
| ENSOARG00000020529 | *CAPN3* | 132361,50 | protein_coding |
| ENSOARG00000010995 | *PPDPF* | 129639,84 | protein_coding |
| ENSOARG00000002910 | *ATP5A1* | 128784,68 | protein_coding |
| ENSOARG00000020946 | *TMOD4* | 125828,24 | protein_coding |
| ENSOARG00000011805 | *RPLP0* | 122423,75 | protein_coding |
| ENSOARG00000001499 | *COQ8A* | 122334,35 | protein_coding |
| ENSOARG00000017957 | *GPD1* | 118860,94 | protein_coding |
| ENSOARG00000016955 | *SLN* | 118205,02 | protein_coding |
| ENSOARG00000005428 | *TPI1* | 115203,34 | protein_coding |
| ENSOARG00000015514 | *MYOT* | 114010,00 | protein_coding |
| ENSOARG00000001573 | *MYLK2* | 110413,80 | protein_coding |
| ENSOARG00000018207 |  | 109663,11 | protein_coding |
| ENSOARG00000002247 |  | 107344,26 | pseudogene |
| ENSOARG00000000491 | *SRRM2* | 105585,47 | protein_coding |
| ENSOARG00000004362 | *KIF1C* | 105512,24 | protein_coding |
| ENSOARG00000015170 | *CKMT2* | 103802,99 | protein_coding |
| ENSOARG00000013650 | *MDH2* | 103725,44 | protein_coding |
| ENSOARG00000013191 | *RPS11* | 100646,22 | protein_coding |
| ENSOARG00000015619 |  | 100178,79 | protein_coding |
| ENSOARG00000001717 | *GOT2* | 99688,74 | protein_coding |
| ENSOARG00000012740 | *ZFAND5* | 98001,04 | protein_coding |
| ENSOARG00000015846 | *PLEC* | 96716,14 | protein_coding |
| ENSOARG00000007343 | *NFE2L1* | 96557,82 | protein_coding |
| ENSOARG00000015142 | *EEF1G* | 96418,88 | protein_coding |
| ENSOARG00000020447 | *YBX1* | 95729,58 | protein_coding |
| ENSOARG00000009609 |  | 94555,62 | protein_coding |
| ENSOARG00000004399 | *UBB* | 94357,45 | protein_coding |
| ENSOARG00000016775 | *ATF4* | 94137,73 | protein_coding |
| ENSOARG00000018757 | *GLUL* | 88539,33 | protein_coding |
| ENSOARG00000003903 | *HSP90AA1* | 87662,63 | protein_coding |
| ENSOARG00000020013 | *AMPD1* | 87660,48 | protein_coding |
| ENSOARG00000018803 | *PGK1* | 87462,30 | protein_coding |
| ENSOARG00000016096 | *PPP1R1A* | 87224,28 | protein_coding |
| ENSOARG00000017449 | *CACNA1S* | 86620,07 | protein_coding |
| ENSOARG00000013747 | *ANKRD23* | 85673,36 | protein_coding |
| ENSOARG00000011503 | *COX4I1* | 82640,45 | protein_coding |
| ENSOARG00000015094 | *UQCRC2* | 81895,14 | protein_coding |
| ENSOARG00000016188 | *BIN1* | 81782,05 | protein_coding |
| ENSOARG00000009990 | *SYNM* | 79568,76 | protein_coding |
| ENSOARG00000004431 | *XIRP2* | 78859,00 | protein_coding |
| ENSOARG00000005473 | *GSN* | 78505,73 | protein_coding |
| ENSOARG00000009782 | *NDUFS2* | 78366,79 | protein_coding |
| ENSOARG00000019443 |  | 77974,76 | pseudogene |
| ENSOARG00000018177 | *UBC* | 77947,83 | protein_coding |
| ENSOARG00000014862 | *OGDH* | 77664,57 | protein_coding |
| ENSOARG00000010243 | *EIF4G2* | 76702,78 | protein_coding |
| ENSOARG00000004502 | *GPI* | 73709,72 | protein_coding |
| ENSOARG00000014168 | *ASB2* | 73278,91 | protein_coding |
| ENSOARG00000012970 | *SLC25A3* | 70891,13 | protein_coding |
| ENSOARG00000012220 | *GMPR* | 70001,51 | protein_coding |
| ENSOARG00000013272 |  | 68826,47 | protein_coding |
| ENSOARG00000006729 | *TACC2* | 68353,65 | protein_coding |
| ENSOARG00000014339 |  | 68067,16 | protein_coding |
| ENSOARG00000008601 |  | 67599,73 | protein_coding |
| ENSOARG00000014369 | *SVIL* | 67517,88 | protein_coding |
| ENSOARG00000013571 | *RPS17* | 66915,82 | protein_coding |
| ENSOARG00000000936 | *LMOD2* | 66850,12 | protein_coding |
| ENSOARG00000000142 | *HSPA8* | 66377,31 | protein_coding |
| ENSOARG00000003386 |  | 66072,51 | protein_coding |
| ENSOARG00000008668 | *FBP2* | 65606,15 | protein_coding |
| ENSOARG00000004817 | *SPEG* | 65487,68 | protein_coding |
| ENSOARG00000015048 | *CYC1* | 65413,36 | protein_coding |
| ENSOARG00000011321 | *AK1* | 65116,10 | protein_coding |
| ENSOARG00000003677 |  | 64348,18 | protein_coding |
| ENSOARG00000015194 | *CRYAB* | 63877,52 | protein_coding |
| ENSOARG00000009032 | *SPARC* | 63149,45 | protein_coding |
| ENSOARG00000018755 | *TNNI1* | 63093,44 | protein_coding |
| ENSOARG00000016718 | *UQCRC1* | 62945,89 | protein_coding |
| ENSOARG00000009343 | *HSP90AB1* | 62110,12 | protein_coding |
| ENSOARG00000018519 |  | 61987,33 | protein_coding |
| ENSOARG00000013306 | *ALPK3* | 61920,56 | protein_coding |
| ENSOARG00000001277 | *MYBPH* | 61755,77 | protein_coding |
| ENSOARG00000009775 | *SH3BGR* | 61698,69 | protein_coding |
| ENSOARG00000001947 | *C10orf71* | 60991,08 | protein_coding |
| ENSOARG00000013082 | *PFKFB3* | 60406,26 | protein_coding |
| ENSOARG00000002683 | *SQSTM1* | 60266,24 | protein_coding |
| ENSOARG00000000683 | *PTP4A2* | 59765,42 | protein_coding |
| ENSOARG00000003782 | *B2M* | 58895,18 | protein_coding |
| ENSOARG00000009101 |  | 58749,79 | protein_coding |
| ENSOARG00000010033 | *LMOD3* | 58595,77 | protein_coding |
| ENSOARG00000010582 |  | 57945,24 | protein_coding |
| ENSOARG00000004251 |  | 57654,45 | protein_coding |
| ENSOARG00000003306 | *DHRS7C* | 57253,79 | protein_coding |
| ENSOARG00000012193 | *VCP* | 56759,44 | protein_coding |
| ENSOARG00000011328 | *VIM* | 56594,65 | protein_coding |
| ENSOARG00000018593 | *HADHA* | 56323,24 | protein_coding |
| ENSOARG00000010650 | *ALDH2* | 56161,68 | protein_coding |
| ENSOARG00000012645 | *RPL8* | 55602,71 | protein_coding |
| ENSOARG00000013296 | *NDUFV1* | 55556,39 | protein_coding |
| ENSOARG00000016963 |  | 55343,14 | protein_coding |
| ENSOARG00000003848 | *RPS5* | 55020,03 | protein_coding |
| ENSOARG00000016537 | *HSPA9* | 54931,72 | protein_coding |
| ENSOARG00000004471 | *UQCRFS1* | 54860,63 | protein_coding |
| ENSOARG00000001136 |  | 54551,53 | protein_coding |
| ENSOARG00000016799 | *RPL3L* | 54344,74 | protein_coding |
| ENSOARG00000006115 | *COL4A1* | 53536,96 | protein_coding |
| ENSOARG00000007513 | *SYNPO* | 53268,78 | protein_coding |
| ENSOARG00000016476 | *COL3A1* | 53143,85 | protein_coding |
| ENSOARG00000015002 | *HLA-E* | 52839,05 | protein_coding |
| ENSOARG00000017889 | *PHKA1* | 52774,43 | protein_coding |
| ENSOARG00000020060 | *PABPC4* | 52622,57 | protein_coding |
| ENSOARG00000008297 | *ATP1A2* | 52321,00 | protein_coding |
| ENSOARG00000006966 | *UNC45B* | 51876,19 | protein_coding |
| ENSOARG00000008276 | *NACA2* | 51836,34 | protein_coding |
| ENSOARG00000002647 | *MAP4* | 51641,39 | protein_coding |
| ENSOARG00000013670 | *COX5B* | 51056,57 | protein_coding |
| ENSOARG00000011523 | *RPS3* | 50798,08 | protein_coding |
| ENSOARG00000005380 | *PDLIM7* | 50402,81 | protein_coding |
| ENSOARG00000002458 |  | 49746,90 | protein_coding |
| ENSOARG00000013169 |  | 49688,74 | protein_coding |
| ENSOARG00000007328 | *RPS14* | 49627,35 | protein_coding |
| ENSOARG00000020596 | *EIF4G1* | 49376,40 | protein_coding |
| ENSOARG00000019120 | *MYO18B* | 49256,85 | protein_coding |
| ENSOARG00000007288 |  | 49014,52 | protein_coding |
| ENSOARG00000014559 | *LAMB2* | 48971,44 | protein_coding |
| ENSOARG00000000606 | *AC006254.1* | 48764,65 | protein_coding |
| ENSOARG00000013705 | *VDAC1* | 48716,18 | protein_coding |
| ENSOARG00000006341 | *IDH3B* | 48158,28 | protein_coding |
| ENSOARG00000020069 | *MDH1* | 47702,70 | protein_coding |
| ENSOARG00000012463 | *GYS1* | 47588,53 | protein_coding |
| ENSOARG00000006957 | *PSAP* | 47075,87 | protein_coding |
| ENSOARG00000010640 | *CALM3* | 46706,45 | protein_coding |
| ENSOARG00000003064 | *DYNC1H1* | 46668,75 | protein_coding |
| ENSOARG00000012014 |  | 45549,72 | protein_coding |
| RIPOLLESA | | | |
| **Gene Ensembl ID** | **Gene name** | **Normalized DESeq2 counts** | **Gene type** |
| ENSOARG00000003044 | *ACTA1* | 7214440,33 | protein_coding |
| ENSOARG00000009785 | *CKM* | 5094719,67 | protein_coding |
| ENSOARG00000004143 | *ALDOA* | 2569599,72 | protein_coding |
| ENSOARG00000007894 | *GAPDH* | 2542389,08 | protein_coding |
| ENSOARG00000011486 |  | 2389901,89 | protein_coding |
| ENSOARG00000006071 | *MYLPF* | 2111536,21 | protein_coding |
| ENSOARG00000020797 | *TPM1* | 1690957,83 | protein_coding |
| ENSOARG00000011889 | *TPM2* | 1594570,90 | protein_coding |
| ENSOARG00000002132 | *ATP2A1* | 1512547,44 | protein_coding |
| ENSOARG00000008997 | *PYGM* | 1293626,94 | protein_coding |
| ENSOARG00000005506 | *ENO3* | 1290464,22 | protein_coding |
| ENSOARG00000019316 | *MYH7* | 1136270,95 | protein_coding |
| ENSOARG00000012656 | *MYH2* | 1077205,38 | protein_coding |
| ENSOARG00000003852 | *TNNI2* | 1067998,14 | protein_coding |
| ENSOARG00000018639 | *MB* | 1028787,40 | protein_coding |
| ENSOARG00000013964 | *MYBPC2* | 1001090,85 | protein_coding |
| ENSOARG00000017195 | *TTN* | 977145,97 | protein_coding |
| ENSOARG00000020185 | *DES* | 942166,28 | protein_coding |
| ENSOARG00000005704 | *RYR1* | 864342,42 | protein_coding |
| ENSOARG00000000035 | *MT-CYB* | 792195,39 | protein_coding |
| ENSOARG00000003372 | *FLNC* | 778857,96 | protein_coding |
| ENSOARG00000018886 | *PKM* | 709818,34 | protein_coding |
| ENSOARG00000006566 | *TNNC2* | 691215,14 | protein_coding |
| ENSOARG00000006670 | *ACTN3* | 685306,20 | protein_coding |
| ENSOARG00000008405 | *CASQ1* | 641638,81 | protein_coding |
| ENSOARG00000013633 | *PGAM2* | 577287,94 | protein_coding |
| ENSOARG00000019190 | *MYL1* | 561288,89 | protein_coding |
| ENSOARG00000011052 |  | 518892,65 | protein_coding |
| ENSOARG00000003713 | *ACTN2* | 480868,47 | protein_coding |
| ENSOARG00000007876 | *MYOZ1* | 463328,19 | protein_coding |
| ENSOARG00000009602 | *LDHA* | 452461,51 | protein_coding |
| ENSOARG00000017779 | *MYL2* | 437259,65 | protein_coding |
| ENSOARG00000019300 | *PFKM* | 388723,45 | protein_coding |
| ENSOARG00000000016 | *MT-CO1* | 381673,49 | protein_coding |
| ENSOARG00000008306 | *ATP5B* | 351637,45 | protein_coding |
| ENSOARG00000000611 | *LDB3* | 310201,09 | protein_coding |
| ENSOARG00000005748 | *MYOM2* | 304935,32 | protein_coding |
| ENSOARG00000015112 | *MYBPC1* | 286980,72 | protein_coding |
| ENSOARG00000013116 | *CA3* | 280972,00 | protein_coding |
| ENSOARG00000003501 | *PDK4* | 260657,29 | protein_coding |
| ENSOARG00000026131 |  | 252978,27 | lincRNA |
| ENSOARG00000004312 | *TNNC1* | 251541,16 | protein_coding |
| ENSOARG00000011424 | *FHL1* | 248007,51 | protein_coding |
| ENSOARG00000017679 |  | 245641,98 | protein_coding |
| ENSOARG00000001823 | *TNNT1* | 237650,59 | protein_coding |
| ENSOARG00000007666 | *SLC25A4* | 232229,72 | protein_coding |
| ENSOARG00000008659 | *NEB* | 231853,36 | protein_coding |
| ENSOARG00000020482 | *PDE4DIP* | 227646,17 | protein_coding |
| ENSOARG00000005438 | *OBSCN* | 223684,10 | protein_coding |
| ENSOARG00000009826 | *PGM1* | 220762,16 | protein_coding |
| ENSOARG00000018307 | *ACO2* | 218577,76 | protein_coding |
| ENSOARG00000005428 | *TPI1* | 218433,51 | protein_coding |
| ENSOARG00000011109 | *EEF2* | 217801,18 | protein_coding |
| ENSOARG00000018001 | *IGFN1* | 213845,62 | protein_coding |
| ENSOARG00000012744 | *NRAP* | 213190,51 | protein_coding |
| ENSOARG00000011575 | *TCAP* | 208257,72 | protein_coding |
| ENSOARG00000004502 | *GPI* | 177257,46 | protein_coding |
| ENSOARG00000004362 | *KIF1C* | 175633,80 | protein_coding |
| ENSOARG00000018803 | *PGK1* | 173568,71 | protein_coding |
| ENSOARG00000020020 | *TUBA4A* | 169582,77 | protein_coding |
| ENSOARG00000011805 | *RPLP0* | 165059,96 | protein_coding |
| ENSOARG00000002910 | *ATP5A1* | 155543,61 | protein_coding |
| ENSOARG00000000032 | *MT-ND5* | 154518,65 | protein_coding |
| ENSOARG00000010995 | *PPDPF* | 149312,54 | protein_coding |
| ENSOARG00000010213 | *MYOM1* | 147519,68 | protein_coding |
| ENSOARG00000015170 | *CKMT2* | 147210,57 | protein_coding |
| ENSOARG00000015846 | *PLEC* | 143672,58 | protein_coding |
| ENSOARG00000018666 | *RPLP1* | 142313,56 | protein_coding |
| ENSOARG00000002247 |  | 138164,94 | pseudogene |
| ENSOARG00000015142 | *EEF1G* | 138150,84 | protein_coding |
| ENSOARG00000014862 | *OGDH* | 136143,23 | protein_coding |
| ENSOARG00000000022 | *MT-ATP6* | 136137,81 | protein_coding |
| ENSOARG00000020919 | *YBX3* | 134484,86 | protein_coding |
| ENSOARG00000009032 | *SPARC* | 133897,01 | protein_coding |
| ENSOARG00000017957 | *GPD1* | 133234,31 | protein_coding |
| ENSOARG00000016476 | *COL3A1* | 132199,59 | protein_coding |
| ENSOARG00000013306 | *ALPK3* | 129676,80 | protein_coding |
| ENSOARG00000013747 | *ANKRD23* | 129501,09 | protein_coding |
| ENSOARG00000017449 | *CACNA1S* | 128662,69 | protein_coding |
| ENSOARG00000013650 | *MDH2* | 128583,51 | protein_coding |
| ENSOARG00000020946 | *TMOD4* | 128028,19 | protein_coding |
| ENSOARG00000015619 |  | 126184,36 | protein_coding |
| ENSOARG00000000142 | *HSPA8* | 123704,95 | protein_coding |
| ENSOARG00000016562 | *GNAS* | 122939,21 | protein_coding |
| ENSOARG00000016188 | *BIN1* | 119899,06 | protein_coding |
| ENSOARG00000003388 | *KLHL41* | 115472,77 | protein_coding |
| ENSOARG00000014048 | *CCNG1* | 113867,55 | protein_coding |
| ENSOARG00000020658 | *TXNIP* | 113622,43 | protein_coding |
| ENSOARG00000001717 | *GOT2* | 113583,39 | protein_coding |
| ENSOARG00000007924 | *PDLIM3* | 112374,05 | protein_coding |
| ENSOARG00000001845 | *TPM3* | 111756,91 | protein_coding |
| ENSOARG00000016096 | *PPP1R1A* | 111545,41 | protein_coding |
| ENSOARG00000016226 | *ATP2A2* | 110522,62 | protein_coding |
| ENSOARG00000011321 | *AK1* | 109656,02 | protein_coding |
| ENSOARG00000014988 | *AHNAK* | 109043,22 | protein_coding |
| ENSOARG00000015194 | *CRYAB* | 107101,77 | protein_coding |
| ENSOARG00000001499 | *COQ8A* | 105524,75 | protein_coding |
| ENSOARG00000001277 | *MYBPH* | 105145,14 | protein_coding |
| ENSOARG00000013191 | *RPS11* | 103509,55 | protein_coding |
| ENSOARG00000019443 |  | 103219,96 | pseudogene |
| ENSOARG00000018207 |  | 100889,14 | protein_coding |
| ENSOARG00000011503 | *COX4I1* | 100761,15 | protein_coding |
| ENSOARG00000005473 | *GSN* | 100333,82 | protein_coding |
| ENSOARG00000003306 | *DHRS7C* | 99516,02 | protein_coding |
| ENSOARG00000006115 | *COL4A1* | 98175,45 | protein_coding |
| ENSOARG00000020529 | *CAPN3* | 98081,09 | protein_coding |
| ENSOARG00000020447 | *YBX1* | 94814,25 | protein_coding |
| ENSOARG00000009782 | *NDUFS2* | 93296,88 | protein_coding |
| ENSOARG00000008668 | *FBP2* | 92443,29 | protein_coding |
| ENSOARG00000010069 | *MYL6* | 90674,30 | protein_coding |
| ENSOARG00000019120 | *MYO18B* | 90459,55 | protein_coding |
| ENSOARG00000001573 | *MYLK2* | 90227,44 | protein_coding |
| ENSOARG00000004399 | *UBB* | 88282,74 | protein_coding |
| ENSOARG00000003903 | *HSP90AA1* | 88267,55 | protein_coding |
| ENSOARG00000016718 | *UQCRC1* | 87240,43 | protein_coding |
| ENSOARG00000012970 | *SLC25A3* | 87045,20 | protein_coding |
| ENSOARG00000015048 | *CYC1* | 84829,35 | protein_coding |
| ENSOARG00000016955 | *SLN* | 84534,33 | protein_coding |
| ENSOARG00000003074 | *SRL* | 84530,00 | protein_coding |
| ENSOARG00000005466 | *TRIM63* | 83216,54 | protein_coding |
| ENSOARG00000011328 | *VIM* | 83140,61 | protein_coding |
| ENSOARG00000004871 | *COL1A1* | 83067,94 | protein_coding |
| ENSOARG00000016775 | *ATF4* | 81469,23 | protein_coding |
| ENSOARG00000012463 | *GYS1* | 81097,21 | protein_coding |
| ENSOARG00000000491 | *SRRM2* | 80537,55 | protein_coding |
| ENSOARG00000005380 | *PDLIM7* | 79331,47 | protein_coding |
| ENSOARG00000014559 | *LAMB2* | 78703,48 | protein_coding |
| ENSOARG00000016963 |  | 76086,32 | protein_coding |
| ENSOARG00000013082 | *PFKFB3* | 75944,24 | protein_coding |
| ENSOARG00000017434 | *CMYA5* | 74729,48 | protein_coding |
| ENSOARG00000012645 | *RPL8* | 73394,33 | protein_coding |
| ENSOARG00000006957 | *PSAP* | 73225,13 | protein_coding |
| ENSOARG00000007513 | *SYNPO* | 71605,81 | protein_coding |
| ENSOARG00000014369 | *SVIL* | 71591,71 | protein_coding |
| ENSOARG00000013296 | *NDUFV1* | 71264,16 | protein_coding |
| ENSOARG00000000037 |  | 71038,56 | Mt_tRNA |
| ENSOARG00000001508 | *COL1A2* | 70386,71 | protein_coding |
| ENSOARG00000016799 | *RPL3L* | 69944,19 | protein_coding |
| ENSOARG00000008601 |  | 69848,74 | protein_coding |
| ENSOARG00000013748 | *HSPB1* | 68408,38 | protein_coding |
| ENSOARG00000010650 | *ALDH2* | 68069,99 | protein_coding |
| ENSOARG00000015094 | *UQCRC2* | 67549,38 | protein_coding |
| ENSOARG00000003742 |  | 66141,55 | pseudogene |
| ENSOARG00000003677 |  | 65750,01 | protein_coding |
| ENSOARG00000009609 |  | 65087,32 | protein_coding |
| ENSOARG00000010243 | *EIF4G2* | 64473,43 | protein_coding |
| ENSOARG00000003782 | *B2M* | 64428,96 | protein_coding |
| ENSOARG00000015908 | *ANKRD1* | 64340,02 | protein_coding |
| ENSOARG00000003848 | *RPS5* | 64055,85 | protein_coding |
| ENSOARG00000004471 | *UQCRFS1* | 63824,83 | protein_coding |
| ENSOARG00000011323 | *NMRK2* | 63619,84 | protein_coding |
| ENSOARG00000000033 | *MT-ND6* | 62933,28 | protein_coding |
| ENSOARG00000013272 |  | 62450,63 | protein_coding |
| ENSOARG00000014339 |  | 62440,87 | protein_coding |
| ENSOARG00000013571 | *RPS17* | 62396,40 | protein_coding |
| ENSOARG00000008822 | *CRAT* | 62363,87 | protein_coding |
| ENSOARG00000015514 | *MYOT* | 61145,85 | protein_coding |
| ENSOARG00000013899 | *MYH3* | 60411,57 | protein_coding |
| ENSOARG00000008821 | *SLC25A34* | 60238,03 | protein_coding |
| ENSOARG00000003064 | *DYNC1H1* | 59833,48 | protein_coding |
| ENSOARG00000016878 | *SYNPO2* | 59682,71 | protein_coding |
| ENSOARG00000010640 | *CALM3* | 58807,44 | protein_coding |
| ENSOARG00000009343 | *HSP90AB1* | 58589,43 | protein_coding |
| ENSOARG00000006966 | *UNC45B* | 58547,13 | protein_coding |
| ENSOARG00000018519 |  | 58150,16 | protein_coding |
| ENSOARG00000017870 | *HDLBP* | 58012,42 | protein_coding |
| ENSOARG00000013670 | *COX5B* | 57860,57 | protein_coding |
| ENSOARG00000018506 | *ACTG1* | 56642,56 | protein_coding |
| ENSOARG00000010582 |  | 56565,55 | protein_coding |
| ENSOARG00000014168 | *ASB2* | 56395,27 | protein_coding |
| ENSOARG00000000170 | *ATP5G3* | 56277,05 | protein_coding |
| ENSOARG00000019993 | *ACTC1* | 56053,62 | protein_coding |
| ENSOARG00000011523 | *RPS3* | 55205,45 | protein_coding |
| ENSOARG00000001136 |  | 54984,19 | protein_coding |
| ENSOARG00000002458 |  | 54817,16 | protein_coding |
| ENSOARG00000008758 | *VDAC2* | 54665,32 | protein_coding |
| ENSOARG00000020596 | *EIF4G1* | 54593,73 | protein_coding |
| ENSOARG00000008188 | *ANXA6* | 54330,18 | protein_coding |
| ENSOARG00000006996 | *MYL3* | 54194,60 | protein_coding |
| ENSOARG00000026007 |  | 54075,29 | lincRNA |
| ENSOARG00000015002 | *HLA-E* | 53921,28 | protein_coding |
| ENSOARG00000019213 | *SYPL2* | 53854,03 | protein_coding |
| ENSOARG00000004251 |  | 53798,72 | protein_coding |
| ENSOARG00000006653 | *ATP5G1* | 53743,40 | protein_coding |
| ENSOARG00000007702 | *ADSSL1* | 53596,98 | protein_coding |
| ENSOARG00000005289 | *COX7A1* | 53278,11 | protein_coding |
| ENSOARG00000020069 | *MDH1* | 52652,29 | protein_coding |
| ENSOARG00000006515 | *COL4A2* | 52327,99 | protein_coding |
| ENSOARG00000007288 |  | 51876,79 | protein_coding |
| ENSOARG00000012193 | *VCP* | 51750,98 | protein_coding |
| ENSOARG00000009101 |  | 51677,22 | protein_coding |
| ENSOARG00000018755 | *TNNI1* | 51149,02 | protein_coding |
| ENSOARG00000013169 |  | 50338,82 | protein_coding |
| ENSOARG00000006456 | *RPL29* | 49678,29 | protein_coding |
| ENSOARG00000009418 |  | 49577,42 | protein_coding |
| ENSOARG00000007328 | *RPS14* | 49400,63 | protein_coding |
| ENSOARG00000008297 | *ATP1A2* | 49237,94 | protein_coding |
| **GALLEGA** | | | |
| **Gene Ensembl ID** | **Gene name** | **Normalized DESeq2 counts** | **Gene type** |
| ENSOARG00000003044 | *ACTA1* | 12126628,52 | protein_coding |
| ENSOARG00000009785 | *CKM* | 8709202,20 | protein_coding |
| ENSOARG00000004143 | *ALDOA* | 4183403,47 | protein_coding |
| ENSOARG00000007894 | *GAPDH* | 4164298,07 | protein_coding |
| ENSOARG00000011889 | *TPM2* | 3355183,87 | protein_coding |
| ENSOARG00000019316 | *MYH7* | 3270982,71 | protein_coding |
| ENSOARG00000006071 | *MYLPF* | 3224258,69 | protein_coding |
| ENSOARG00000018639 | *MB* | 2922463,55 | protein_coding |
| ENSOARG00000002132 | *ATP2A1* | 2594051,62 | protein_coding |
| ENSOARG00000020185 | *DES* | 2529055,29 | protein_coding |
| ENSOARG00000008997 | *PYGM* | 2399388,03 | protein_coding |
| ENSOARG00000005506 | *ENO3* | 1977996,58 | protein_coding |
| ENSOARG00000020797 | *TPM1* | 1870925,28 | protein_coding |
| ENSOARG00000011486 |  | 1808101,32 | protein_coding |
| ENSOARG00000003852 | *TNNI2* | 1543497,09 | protein_coding |
| ENSOARG00000013964 | *MYBPC2* | 1423641,65 | protein_coding |
| ENSOARG00000012656 | *MYH2* | 1414278,77 | protein_coding |
| ENSOARG00000005704 | *RYR1* | 1203450,47 | protein_coding |
| ENSOARG00000011052 |  | 1188002,84 | protein_coding |
| ENSOARG00000017779 | *MYL2* | 1163023,61 | protein_coding |
| ENSOARG00000000035 | *MT-CYB* | 1089604,45 | protein_coding |
| ENSOARG00000006670 | *ACTN3* | 1030370,48 | protein_coding |
| ENSOARG00000013633 | *PGAM2* | 1027717,53 | protein_coding |
| ENSOARG00000006566 | *TNNC2* | 900977,41 | protein_coding |
| ENSOARG00000008405 | *CASQ1* | 865080,58 | protein_coding |
| ENSOARG00000000016 | *MT-CO1* | 826209,93 | protein_coding |
| ENSOARG00000003372 | *FLNC* | 781314,80 | protein_coding |
| ENSOARG00000003713 | *ACTN2* | 769648,49 | protein_coding |
| ENSOARG00000004312 | *TNNC1* | 768640,64 | protein_coding |
| ENSOARG00000007876 | *MYOZ1* | 768639,13 | protein_coding |
| ENSOARG00000001823 | *TNNT1* | 713642,93 | protein_coding |
| ENSOARG00000018886 | *PKM* | 713124,70 | protein_coding |
| ENSOARG00000019190 | *MYL1* | 622972,88 | protein_coding |
| ENSOARG00000017195 | *TTN* | 589510,55 | protein_coding |
| ENSOARG00000011575 | *TCAP* | 505310,90 | protein_coding |
| ENSOARG00000008306 | *ATP5B* | 492329,41 | protein_coding |
| ENSOARG00000011424 | *FHL1* | 425614,17 | protein_coding |
| ENSOARG00000013116 | *CA3* | 410128,88 | protein_coding |
| ENSOARG00000017679 |  | 402370,42 | protein_coding |
| ENSOARG00000005438 | *OBSCN* | 394953,93 | protein_coding |
| ENSOARG00000000611 | *LDB3* | 392090,08 | protein_coding |
| ENSOARG00000026131 |  | 388400,67 | lincRNA |
| ENSOARG00000015112 | *MYBPC1* | 379348,12 | protein_coding |
| ENSOARG00000009602 | *LDHA* | 373701,77 | protein_coding |
| ENSOARG00000005748 | *MYOM2* | 363915,56 | protein_coding |
| ENSOARG00000012744 | *NRAP* | 363225,59 | protein_coding |
| ENSOARG00000018307 | *ACO2* | 351132,93 | protein_coding |
| ENSOARG00000007666 | *SLC25A4* | 340222,88 | protein_coding |
| ENSOARG00000011109 | *EEF2* | 331384,26 | protein_coding |
| ENSOARG00000004362 | *KIF1C* | 329416,77 | protein_coding |
| ENSOARG00000019300 | *PFKM* | 326391,72 | protein_coding |
| ENSOARG00000010995 | *PPDPF* | 311212,26 | protein_coding |
| ENSOARG00000020020 | *TUBA4A* | 304562,58 | protein_coding |
| ENSOARG00000005428 | *TPI1* | 301572,18 | protein_coding |
| ENSOARG00000020482 | *PDE4DIP* | 294948,11 | protein_coding |
| ENSOARG00000011805 | *RPLP0* | 289324,35 | protein_coding |
| ENSOARG00000018666 | *RPLP1* | 270771,84 | protein_coding |
| ENSOARG00000000022 | *MT-ATP6* | 260970,56 | protein_coding |
| ENSOARG00000015194 | *CRYAB* | 247597,38 | protein_coding |
| ENSOARG00000015142 | *EEF1G* | 246806,47 | protein_coding |
| ENSOARG00000016226 | *ATP2A2* | 233376,05 | protein_coding |
| ENSOARG00000010069 | *MYL6* | 232124,15 | protein_coding |
| ENSOARG00000020946 | *TMOD4* | 228345,85 | protein_coding |
| ENSOARG00000020919 | *YBX3* | 224508,80 | protein_coding |
| ENSOARG00000015846 | *PLEC* | 212208,25 | protein_coding |
| ENSOARG00000015619 |  | 211030,17 | protein_coding |
| ENSOARG00000011503 | *COX4I1* | 210777,08 | protein_coding |
| ENSOARG00000009826 | *PGM1* | 208259,72 | protein_coding |
| ENSOARG00000013650 | *MDH2* | 207736,97 | protein_coding |
| ENSOARG00000017957 | *GPD1* | 207223,25 | protein_coding |
| ENSOARG00000018755 | *TNNI1* | 205430,52 | protein_coding |
| ENSOARG00000017449 | *CACNA1S* | 201581,42 | protein_coding |
| ENSOARG00000013191 | *RPS11* | 196207,74 | protein_coding |
| ENSOARG00000016096 | *PPP1R1A* | 188944,92 | protein_coding |
| ENSOARG00000014862 | *OGDH* | 187412,81 | protein_coding |
| ENSOARG00000019443 |  | 180645,62 | pseudogene |
| ENSOARG00000001845 | *TPM3* | 179554,92 | protein_coding |
| ENSOARG00000015170 | *CKMT2* | 179327,44 | protein_coding |
| ENSOARG00000000037 |  | 178452,16 | Mt_tRNA |
| ENSOARG00000009032 | *SPARC* | 177807,38 | protein_coding |
| ENSOARG00000018001 | *IGFN1* | 176410,86 | protein_coding |
| ENSOARG00000001573 | *MYLK2* | 174004,98 | protein_coding |
| ENSOARG00000011321 | *AK1* | 172549,71 | protein_coding |
| ENSOARG00000016188 | *BIN1* | 171740,72 | protein_coding |
| ENSOARG00000016718 | *UQCRC1* | 169051,62 | protein_coding |
| ENSOARG00000015048 | *CYC1* | 169033,54 | protein_coding |
| ENSOARG00000007924 | *PDLIM3* | 167679,20 | protein_coding |
| ENSOARG00000010213 | *MYOM1* | 167655,09 | protein_coding |
| ENSOARG00000001717 | *GOT2* | 166035,61 | protein_coding |
| ENSOARG00000000032 | *MT-ND5* | 163337,47 | protein_coding |
| ENSOARG00000016562 | *GNAS* | 162718,30 | protein_coding |
| ENSOARG00000004502 | *GPI* | 162308,53 | protein_coding |
| ENSOARG00000004399 | *UBB* | 161457,36 | protein_coding |
| ENSOARG00000007513 | *SYNPO* | 159379,90 | protein_coding |
| ENSOARG00000002910 | *ATP5A1* | 159324,16 | protein_coding |
| ENSOARG00000020658 | *TXNIP* | 153813,40 | protein_coding |
| ENSOARG00000015002 | *HLA-E* | 152380,72 | protein_coding |
| ENSOARG00000001499 | *COQ8A* | 151198,12 | protein_coding |
| ENSOARG00000006115 | *COL4A1* | 150777,80 | protein_coding |
| ENSOARG00000008821 | *SLC25A34* | 148744,03 | protein_coding |
| ENSOARG00000008601 |  | 147823,56 | protein_coding |
| ENSOARG00000019120 | *MYO18B* | 146116,70 | protein_coding |
| ENSOARG00000013747 | *ANKRD23* | 143513,47 | protein_coding |
| ENSOARG00000016963 |  | 143344,74 | protein_coding |
| ENSOARG00000009782 | *NDUFS2* | 142945,52 | protein_coding |
| ENSOARG00000014168 | *ASB2* | 139299,80 | protein_coding |
| ENSOARG00000003848 | *RPS5* | 138475,74 | protein_coding |
| ENSOARG00000018803 | *PGK1* | 136554,96 | protein_coding |
| ENSOARG00000016775 | *ATF4* | 134857,14 | protein_coding |
| ENSOARG00000014988 | *AHNAK* | 133996,93 | protein_coding |
| ENSOARG00000013296 | *NDUFV1* | 133575,11 | protein_coding |
| ENSOARG00000001277 | *MYBPH* | 131902,90 | protein_coding |
| ENSOARG00000003306 | *DHRS7C* | 131330,43 | protein_coding |
| ENSOARG00000001603 | *FABP3* | 130220,14 | protein_coding |
| ENSOARG00000012645 | *RPL8* | 128739,25 | protein_coding |
| ENSOARG00000013748 | *HSPB1* | 127270,42 | protein_coding |
| ENSOARG00000020529 | *CAPN3* | 126669,32 | protein_coding |
| ENSOARG00000020447 | *YBX1* | 125040,80 | protein_coding |
| ENSOARG00000005473 | *GSN* | 124942,88 | protein_coding |
| ENSOARG00000014048 | *CCNG1* | 124837,42 | protein_coding |
| ENSOARG00000016799 | *RPL3L* | 123936,54 | protein_coding |
| ENSOARG00000003074 | *SRL* | 122729,83 | protein_coding |
| ENSOARG00000013272 |  | 122175,44 | protein_coding |
| ENSOARG00000000606 | *AC006254.1* | 120676,48 | protein_coding |
| ENSOARG00000012463 | *GYS1* | 120575,54 | protein_coding |
| ENSOARG00000012970 | *SLC25A3* | 120348,06 | protein_coding |
| ENSOARG00000005380 | *PDLIM7* | 120346,55 | protein_coding |
| ENSOARG00000013306 | *ALPK3* | 117445,04 | protein_coding |
| ENSOARG00000014339 |  | 114636,93 | protein_coding |
| ENSOARG00000012014 |  | 114209,08 | protein_coding |
| ENSOARG00000008668 | *FBP2* | 113549,24 | protein_coding |
| ENSOARG00000016955 | *SLN* | 112779,42 | protein_coding |
| ENSOARG00000014559 | *LAMB2* | 110572,40 | protein_coding |
| ENSOARG00000010582 |  | 110497,07 | protein_coding |
| ENSOARG00000007343 | *NFE2L1* | 110394,63 | protein_coding |
| ENSOARG00000018207 |  | 109126,16 | protein_coding |
| ENSOARG00000019952 | *FHL3* | 108714,89 | protein_coding |
| ENSOARG00000002458 |  | 105805,84 | protein_coding |
| ENSOARG00000010650 | *ALDH2* | 105335,81 | protein_coding |
| ENSOARG00000003677 |  | 104981,79 | protein_coding |
| ENSOARG00000011523 | *RPS3* | 104594,62 | protein_coding |
| ENSOARG00000011366 | *ATP5D* | 102146,56 | protein_coding |
| ENSOARG00000010640 | *CALM3* | 102072,74 | protein_coding |
| ENSOARG00000006957 | *PSAP* | 100638,55 | protein_coding |
| ENSOARG00000005289 | *COX7A1* | 100579,80 | protein_coding |
| ENSOARG00000004251 |  | 100528,58 | protein_coding |
| ENSOARG00000002753 | *RPS9* | 100114,29 | protein_coding |
| ENSOARG00000004817 | *SPEG* | 99822,03 | protein_coding |
| ENSOARG00000000142 | *HSPA8* | 98665,04 | protein_coding |
| ENSOARG00000006456 | *RPL29* | 98354,70 | protein_coding |
| ENSOARG00000003742 |  | 98205,56 | pseudogene |
| ENSOARG00000000936 | *LMOD2* | 97416,15 | protein_coding |
| ENSOARG00000000820 | *APOBEC2* | 96730,70 | protein_coding |
| ENSOARG00000003782 | *B2M* | 96424,88 | protein_coding |
| ENSOARG00000011328 | *VIM* | 95786,12 | protein_coding |
| ENSOARG00000001947 | *C10orf71* | 95728,88 | protein_coding |
| ENSOARG00000018519 |  | 95246,80 | protein_coding |
| ENSOARG00000009432 | *COX6A2* | 94695,42 | protein_coding |
| ENSOARG00000009343 | *HSP90AB1* | 94383,58 | protein_coding |
| ENSOARG00000011540 |  | 93755,37 | protein_coding |
| ENSOARG00000019706 | *NDRG2* | 93160,30 | protein_coding |
| ENSOARG00000013670 | *COX5B* | 91953,59 | protein_coding |
| ENSOARG00000013571 | *RPS17* | 91864,71 | protein_coding |
| ENSOARG00000010662 | *ACADVL* | 91525,75 | protein_coding |
| ENSOARG00000017870 | *HDLBP* | 90653,49 | protein_coding |
| ENSOARG00000004871 | *COL1A1* | 90605,28 | protein_coding |
| ENSOARG00000006653 | *ATP5G1* | 90368,76 | protein_coding |
| ENSOARG00000004471 | *UQCRFS1* | 89053,59 | protein_coding |
| ENSOARG00000008822 | *CRAT* | 86885,74 | protein_coding |
| ENSOARG00000013169 |  | 86120,44 | protein_coding |
| ENSOARG00000007328 | *RPS14* | 85989,37 | protein_coding |
| ENSOARG00000001136 |  | 85350,62 | protein_coding |
| ENSOARG00000000129 | *EEF1D* | 85061,37 | protein_coding |
| ENSOARG00000007702 | *ADSSL1* | 84987,55 | protein_coding |
| ENSOARG00000014949 | *RPL32* | 84351,81 | protein_coding |
| ENSOARG00000015094 | *UQCRC2* | 82087,54 | protein_coding |
| ENSOARG00000014369 | *SVIL* | 81860,06 | protein_coding |
| ENSOARG00000007183 | *BAG6* | 81286,09 | protein_coding |
| ENSOARG00000016476 | *COL3A1* | 81274,03 | protein_coding |
| ENSOARG00000003386 |  | 81215,28 | protein_coding |
| ENSOARG00000020397 | *SUCLG1* | 81213,77 | protein_coding |
| ENSOARG00000006515 | *COL4A2* | 80769,36 | protein_coding |
| ENSOARG00000011932 | *RPL18* | 80581,05 | protein_coding |
| ENSOARG00000008188 | *ANXA6* | 79876,00 | protein_coding |
| ENSOARG00000011589 | *NDUFS7* | 78841,04 | protein_coding |
| ENSOARG00000017434 | *CMYA5* | 78740,11 | protein_coding |
| ENSOARG00000006966 | *UNC45B* | 78016,99 | protein_coding |
| ENSOARG00000008953 | *PLEKHM2* | 78012,47 | protein_coding |
| ENSOARG00000008248 | *HSPG2* | 77976,31 | protein_coding |
| ENSOARG00000016878 | *SYNPO2* | 77867,84 | protein_coding |
| ENSOARG00000008659 | *NEB* | 77507,79 | protein_coding |
| ENSOARG00000009101 |  | 77441,50 | protein_coding |
| ENSOARG00000020596 | *EIF4G1* | 77101,04 | protein_coding |
| ENSOARG00000003064 | *DYNC1H1* | 76552,67 | protein_coding |
| ENSOARG00000011129 | *SERF2* | 76133,86 | protein_coding |
| ENSOARG00000008758 | *VDAC2* | 75335,42 | protein_coding |
| ENSOARG00000018593 | *HADHA* | 75169,71 | protein_coding |
| **ROJA MALLORQUINA** | | | |
| **Gene Ensembl ID** | **Gene name** | **Normalized DESeq2 counts** | **Gene type** |
| ENSOARG00000003044 | *ACTA1* | 2019923,78 | protein_coding |
| ENSOARG00000012656 | *MYH2* | 1620385,50 | protein_coding |
| ENSOARG00000000035 | *MT-CYB* | 1555377,49 | protein_coding |
| ENSOARG00000019316 | *MYH7* | 1334705,21 | protein_coding |
| ENSOARG00000017779 | *MYL2* | 1229925,57 | protein_coding |
| ENSOARG00000018639 | *MB* | 1204939,99 | protein_coding |
| ENSOARG00000017195 | *TTN* | 1169949,86 | protein_coding |
| ENSOARG00000009785 | *CKM* | 1168356,03 | protein_coding |
| ENSOARG00000011889 | *TPM2* | 1059813,65 | protein_coding |
| ENSOARG00000020185 | *DES* | 678013,83 | protein_coding |
| ENSOARG00000015112 | *MYBPC1* | 577905,48 | protein_coding |
| ENSOARG00000000016 | *MT-CO1* | 575802,98 | protein_coding |
| ENSOARG00000006071 | *MYLPF* | 552926,26 | protein_coding |
| ENSOARG00000004312 | *TNNC1* | 469458,48 | protein_coding |
| ENSOARG00000001823 | *TNNT1* | 432329,11 | protein_coding |
| ENSOARG00000008659 | *NEB* | 430844,78 | protein_coding |
| ENSOARG00000004143 | *ALDOA* | 400262,42 | protein_coding |
| ENSOARG00000000032 | *MT-ND5* | 394228,33 | protein_coding |
| ENSOARG00000007894 | *GAPDH* | 386182,16 | protein_coding |
| ENSOARG00000003713 | *ACTN2* | 356518,23 | protein_coding |
| ENSOARG00000002132 | *ATP2A1* | 338979,01 | protein_coding |
| ENSOARG00000000022 | *MT-ATP6* | 332107,73 | protein_coding |
| ENSOARG00000016226 | *ATP2A2* | 329448,51 | protein_coding |
| ENSOARG00000019190 | *MYL1* | 297005,97 | protein_coding |
| ENSOARG00000011052 |  | 269657,90 | protein_coding |
| ENSOARG00000011486 |  | 253593,12 | protein_coding |
| ENSOARG00000020797 | *TPM1* | 253563,45 | protein_coding |
| ENSOARG00000005704 | *RYR1* | 253160,75 | protein_coding |
| ENSOARG00000020482 | *PDE4DIP* | 246420,17 | protein_coding |
| ENSOARG00000001845 | *TPM3* | 240424,22 | protein_coding |
| ENSOARG00000011575 | *TCAP* | 231805,80 | protein_coding |
| ENSOARG00000011424 | *FHL1* | 229214,41 | protein_coding |
| ENSOARG00000008997 | *PYGM* | 215100,23 | protein_coding |
| ENSOARG00000007666 | *SLC25A4* | 214850,85 | protein_coding |
| ENSOARG00000003388 | *KLHL41* | 211535,31 | protein_coding |
| ENSOARG00000008306 | *ATP5B* | 210759,59 | protein_coding |
| ENSOARG00000003852 | *TNNI2* | 210520,09 | protein_coding |
| ENSOARG00000006566 | *TNNC2* | 203029,92 | protein_coding |
| ENSOARG00000015514 | *MYOT* | 188241,05 | protein_coding |
| ENSOARG00000007876 | *MYOZ1* | 173517,89 | protein_coding |
| ENSOARG00000026131 |  | 171855,53 | lincRNA |
| ENSOARG00000005506 | *ENO3* | 169890,79 | protein_coding |
| ENSOARG00000013964 | *MYBPC2* | 165266,13 | protein_coding |
| ENSOARG00000000611 | *LDB3* | 163728,11 | protein_coding |
| ENSOARG00000008405 | *CASQ1* | 159183,29 | protein_coding |
| ENSOARG00000000033 | *MT-ND6* | 148426,33 | protein_coding |
| ENSOARG00000010213 | *MYOM1* | 142691,08 | protein_coding |
| ENSOARG00000014048 | *CCNG1* | 142214,91 | protein_coding |
| ENSOARG00000010069 | *MYL6* | 135256,73 | protein_coding |
| ENSOARG00000002910 | *ATP5A1* | 135090,70 | protein_coding |
| ENSOARG00000016562 | *GNAS* | 134329,11 | protein_coding |
| ENSOARG00000003372 | *FLNC* | 130341,00 | protein_coding |
| ENSOARG00000009602 | *LDHA* | 127606,90 | protein_coding |
| ENSOARG00000013116 | *CA3* | 123092,45 | protein_coding |
| ENSOARG00000016476 | *COL3A1* | 118732,02 | protein_coding |
| ENSOARG00000017679 |  | 117114,17 | protein_coding |
| ENSOARG00000004431 | *XIRP2* | 115629,84 | protein_coding |
| ENSOARG00000012744 | *NRAP* | 115103,51 | protein_coding |
| ENSOARG00000017434 | *CMYA5* | 113973,84 | protein_coding |
| ENSOARG00000005438 | *OBSCN* | 111484,18 | protein_coding |
| ENSOARG00000020658 | *TXNIP* | 108277,44 | protein_coding |
| ENSOARG00000015170 | *CKMT2* | 107782,90 | protein_coding |
| ENSOARG00000011109 | *EEF2* | 105294,66 | protein_coding |
| ENSOARG00000003903 | *HSP90AA1* | 100104,82 | protein_coding |
| ENSOARG00000007924 | *PDLIM3* | 98359,80 | protein_coding |
| ENSOARG00000019300 | *PFKM* | 96343,48 | protein_coding |
| ENSOARG00000004362 | *KIF1C* | 95929,48 | protein_coding |
| ENSOARG00000014369 | *SVIL* | 94592,81 | protein_coding |
| ENSOARG00000005748 | *MYOM2* | 91026,47 | protein_coding |
| ENSOARG00000018307 | *ACO2* | 90817,35 | protein_coding |
| ENSOARG00000013633 | *PGAM2* | 86733,16 | protein_coding |
| ENSOARG00000010995 | *PPDPF* | 86171,50 | protein_coding |
| ENSOARG00000006867 | *MYOM3* | 85520,83 | protein_coding |
| ENSOARG00000018207 |  | 84545,88 | protein_coding |
| ENSOARG00000009032 | *SPARC* | 84113,51 | protein_coding |
| ENSOARG00000015194 | *CRYAB* | 83072,15 | protein_coding |
| ENSOARG00000000037 |  | 81583,58 | Mt_tRNA |
| ENSOARG00000006670 | *ACTN3* | 79649,22 | protein_coding |
| ENSOARG00000020447 | *YBX1* | 78627,64 | protein_coding |
| ENSOARG00000011805 | *RPLP0* | 77443,57 | protein_coding |
| ENSOARG00000015142 | *EEF1G* | 75746,59 | protein_coding |
| ENSOARG00000018886 | *PKM* | 75307,16 | protein_coding |
| ENSOARG00000018666 | *RPLP1* | 74955,33 | protein_coding |
| ENSOARG00000020280 |  | 74312,43 | protein_coding |
| ENSOARG00000013747 | *ANKRD23* | 74287,70 | protein_coding |
| ENSOARG00000016717 | *MYOZ2* | 74137,93 | protein_coding |
| ENSOARG00000000142 | *HSPA8* | 73169,33 | protein_coding |
| ENSOARG00000012014 |  | 69887,00 | protein_coding |
| ENSOARG00000014988 | *AHNAK* | 69345,13 | protein_coding |
| ENSOARG00000001499 | *COQ8A* | 68902,87 | protein_coding |
| ENSOARG00000015619 |  | 68669,02 | protein_coding |
| ENSOARG00000020919 | *YBX3* | 68069,21 | protein_coding |
| ENSOARG00000013650 | *MDH2* | 67676,41 | protein_coding |
| ENSOARG00000013191 | *RPS11* | 67001,71 | protein_coding |
| ENSOARG00000015094 | *UQCRC2* | 66890,79 | protein_coding |
| ENSOARG00000012970 | *SLC25A3* | 65466,52 | protein_coding |
| ENSOARG00000020069 | *MDH1* | 65240,44 | protein_coding |
| ENSOARG00000020529 | *CAPN3* | 64576,35 | protein_coding |
| ENSOARG00000011328 | *VIM* | 63285,60 | protein_coding |
| ENSOARG00000018755 | *TNNI1* | 62881,49 | protein_coding |
| ENSOARG00000001603 | *FABP3* | 62801,65 | protein_coding |
| ENSOARG00000005473 | *GSN* | 62162,28 | protein_coding |
| ENSOARG00000003782 | *B2M* | 62152,39 | protein_coding |
| ENSOARG00000001717 | *GOT2* | 61067,23 | protein_coding |
| ENSOARG00000003501 | *PDK4* | 60816,43 | protein_coding |
| ENSOARG00000016188 | *BIN1* | 60408,08 | protein_coding |
| ENSOARG00000020946 | *TMOD4* | 60115,59 | protein_coding |
| ENSOARG00000014862 | *OGDH* | 60013,15 | protein_coding |
| ENSOARG00000010243 | *EIF4G2* | 59625,29 | protein_coding |
| ENSOARG00000020020 | *TUBA4A* | 59465,63 | protein_coding |
| ENSOARG00000007343 | *NFE2L1* | 58432,74 | protein_coding |
| ENSOARG00000004399 | *UBB* | 57998,96 | protein_coding |
| ENSOARG00000009343 | *HSP90AB1* | 56320,35 | protein_coding |
| ENSOARG00000000491 | *SRRM2* | 55399,09 | protein_coding |
| ENSOARG00000009782 | *NDUFS2* | 54937,76 | protein_coding |
| ENSOARG00000011503 | *COX4I1* | 54451,70 | protein_coding |
| ENSOARG00000015846 | *PLEC* | 52661,46 | protein_coding |
| ENSOARG00000004471 | *UQCRFS1* | 52639,56 | protein_coding |
| ENSOARG00000006115 | *COL4A1* | 51126,27 | protein_coding |
| ENSOARG00000007614 | *ACSL1* | 50609,83 | protein_coding |
| ENSOARG00000008923 | *OXCT1* | 49243,48 | protein_coding |
| ENSOARG00000013306 | *ALPK3* | 47003,92 | protein_coding |
| ENSOARG00000006957 | *PSAP* | 46657,74 | protein_coding |
| ENSOARG00000008821 | *SLC25A34* | 46553,18 | protein_coding |
| ENSOARG00000008601 |  | 46268,47 | protein_coding |
| ENSOARG00000001508 | *COL1A2* | 45848,82 | protein_coding |
| ENSOARG00000013571 | *RPS17* | 45801,48 | protein_coding |
| ENSOARG00000018593 | *HADHA* | 44873,16 | protein_coding |
| ENSOARG00000015048 | *CYC1* | 44640,02 | protein_coding |
| ENSOARG00000013705 | *VDAC1* | 44554,53 | protein_coding |
| ENSOARG00000009990 | *SYNM* | 44493,07 | protein_coding |
| ENSOARG00000016537 | *HSPA9* | 43970,27 | protein_coding |
| ENSOARG00000016718 | *UQCRC1* | 43913,05 | protein_coding |
| ENSOARG00000003386 |  | 43842,40 | protein_coding |
| ENSOARG00000016955 | *SLN* | 43801,42 | protein_coding |
| ENSOARG00000005428 | *TPI1* | 43743,49 | protein_coding |
| ENSOARG00000010132 |  | 43732,89 | protein_coding |
| ENSOARG00000018519 |  | 43357,04 | protein_coding |
| ENSOARG00000000683 | *PTP4A2* | 43314,65 | protein_coding |
| ENSOARG00000010662 | *ACADVL* | 43196,67 | protein_coding |
| ENSOARG00000003677 |  | 43176,18 | protein_coding |
| ENSOARG00000000021 | *MT-ATP8* | 42981,90 | protein_coding |
| ENSOARG00000017957 | *GPD1* | 42743,10 | protein_coding |
| ENSOARG00000012645 | *RPL8* | 42370,08 | protein_coding |
| ENSOARG00000008573 | *NNT* | 41977,98 | protein_coding |
| ENSOARG00000019443 |  | 41883,31 | pseudogene |
| ENSOARG00000001136 |  | 41727,88 | protein_coding |
| ENSOARG00000015002 | *HLA-E* | 41429,04 | protein_coding |
| ENSOARG00000003074 | *SRL* | 41260,19 | protein_coding |
| ENSOARG00000004502 | *GPI* | 41024,93 | protein_coding |
| ENSOARG00000018563 | *NDUFS1* | 40294,42 | protein_coding |
| ENSOARG00000010033 | *LMOD3* | 40013,95 | protein_coding |
| ENSOARG00000000936 | *LMOD2* | 39077,15 | protein_coding |
| ENSOARG00000006624 | *ACSS1* | 38516,20 | protein_coding |
| ENSOARG00000013296 | *NDUFV1* | 38511,96 | protein_coding |
| ENSOARG00000013272 |  | 38336,75 | protein_coding |
| ENSOARG00000006653 | *ATP5G1* | 38230,78 | protein_coding |
| ENSOARG00000009101 |  | 38225,13 | protein_coding |
| ENSOARG00000012773 | *AP000311.1* | 38098,67 | protein_coding |
| ENSOARG00000010582 |  | 38012,47 | protein_coding |
| ENSOARG00000000170 | *ATP5G3* | 37712,22 | protein_coding |
| ENSOARG00000004871 | *COL1A1* | 37693,85 | protein_coding |
| ENSOARG00000017449 | *CACNA1S* | 37450,11 | protein_coding |
| ENSOARG00000016878 | *SYNPO2* | 37414,08 | protein_coding |
| ENSOARG00000002894 | *RTN4* | 37322,94 | protein_coding |
| ENSOARG00000020060 | *PABPC4* | 37189,42 | protein_coding |
| ENSOARG00000012682 | *HRC* | 37170,34 | protein_coding |
| ENSOARG00000013670 | *COX5B* | 37094,04 | protein_coding |
| ENSOARG00000016775 | *ATF4* | 36939,32 | protein_coding |
| ENSOARG00000011523 | *RPS3* | 36391,09 | protein_coding |
| ENSOARG00000008758 | *VDAC2* | 36222,24 | protein_coding |
| ENSOARG00000006341 | *IDH3B* | 36124,74 | protein_coding |
| ENSOARG00000012740 | *ZFAND5* | 35576,51 | protein_coding |
| ENSOARG00000017443 | *PDLIM5* | 35281,20 | protein_coding |
| ENSOARG00000008297 | *ATP1A2* | 35271,31 | protein_coding |
| ENSOARG00000009418 |  | 35062,90 | protein_coding |
| ENSOARG00000001573 | *MYLK2* | 34997,19 | protein_coding |
| ENSOARG00000006729 | *TACC2* | 34477,22 | protein_coding |
| ENSOARG00000003474 | *SPTBN1* | 34292,83 | protein_coding |
| ENSOARG00000007637 | *LMCD1* | 34073,82 | protein_coding |
| ENSOARG00000015737 | *DCN* | 33831,49 | protein_coding |
| ENSOARG00000009775 | *SH3BGR* | 33784,86 | protein_coding |
| ENSOARG00000010650 | *ALDH2* | 33777,80 | protein_coding |
| ENSOARG00000010362 | *MYL12A* | 33777,80 | protein_coding |
| ENSOARG00000007328 | *RPS14* | 33772,85 | protein_coding |
| ENSOARG00000003848 | *RPS5* | 33749,54 | protein_coding |
| ENSOARG00000012193 | *VCP* | 33483,90 | protein_coding |
| ENSOARG00000018803 | *PGK1* | 33403,36 | protein_coding |
| ENSOARG00000014559 | *LAMB2* | 33235,22 | protein_coding |
| ENSOARG00000011188 | *MYH7B* | 33132,07 | protein_coding |
| ENSOARG00000009826 | *PGM1* | 32997,84 | protein_coding |
| ENSOARG00000020231 | *RPS3A* | 32973,11 | protein_coding |
| ENSOARG00000007288 |  | 32762,58 | protein_coding |
| ENSOARG00000019706 | *NDRG2* | 32561,23 | protein_coding |
| ENSOARG00000026007 |  | 32506,83 | lincRNA |
| ENSOARG00000020674 | *FXR1* | 32199,51 | protein_coding |
| ENSOARG00000004946 | *COX6B1* | 32149,35 | protein_coding |
| **CANARIA DE PELO** | | | |
| **Gene Ensembl ID** | **Gene name** | **Normalized DESeq2 counts** | **Gene type** |
| ENSOARG00000003044 | *ACTA1* | 2063336,23 | protein_coding |
| ENSOARG00000017195 | *TTN* | 1623023,93 | protein_coding |
| ENSOARG00000019316 | *MYH7* | 1530530,89 | protein_coding |
| ENSOARG00000009785 | *CKM* | 1274471,79 | protein_coding |
| ENSOARG00000011889 | *TPM2* | 1141913,11 | protein_coding |
| ENSOARG00000000035 | *MT-CYB* | 1088718,75 | protein_coding |
| ENSOARG00000012656 | *MYH2* | 1041219,91 | protein_coding |
| ENSOARG00000011486 |  | 705056,20 | protein_coding |
| ENSOARG00000017779 | *MYL2* | 691870,43 | protein_coding |
| ENSOARG00000018639 | *MB* | 628754,51 | protein_coding |
| ENSOARG00000020185 | *DES* | 617021,15 | protein_coding |
| ENSOARG00000015112 | *MYBPC1* | 565059,12 | protein_coding |
| ENSOARG00000002132 | *ATP2A1* | 544089,64 | protein_coding |
| ENSOARG00000003501 | *PDK4* | 502186,26 | protein_coding |
| ENSOARG00000007894 | *GAPDH* | 474381,59 | protein_coding |
| ENSOARG00000000032 | *MT-ND5* | 439071,09 | protein_coding |
| ENSOARG00000008659 | *NEB* | 434482,67 | protein_coding |
| ENSOARG00000016226 | *ATP2A2* | 432975,43 | protein_coding |
| ENSOARG00000004312 | *TNNC1* | 421220,58 | protein_coding |
| ENSOARG00000005704 | *RYR1* | 417222,76 | protein_coding |
| ENSOARG00000006071 | *MYLPF* | 412028,93 | protein_coding |
| ENSOARG00000003713 | *ACTN2* | 391884,95 | protein_coding |
| ENSOARG00000004143 | *ALDOA* | 385197,22 | protein_coding |
| ENSOARG00000008997 | *PYGM* | 346559,53 | protein_coding |
| ENSOARG00000001823 | *TNNT1* | 332522,32 | protein_coding |
| ENSOARG00000000022 | *MT-ATP6* | 324523,72 | protein_coding |
| ENSOARG00000011424 | *FHL1* | 277652,52 | protein_coding |
| ENSOARG00000011052 |  | 273394,60 | protein_coding |
| ENSOARG00000011575 | *TCAP* | 272140,04 | protein_coding |
| ENSOARG00000003388 | *KLHL41* | 252159,09 | protein_coding |
| ENSOARG00000003372 | *FLNC* | 230708,69 | protein_coding |
| ENSOARG00000017434 | *CMYA5* | 225379,25 | protein_coding |
| ENSOARG00000012744 | *NRAP* | 222771,59 | protein_coding |
| ENSOARG00000003852 | *TNNI2* | 216925,66 | protein_coding |
| ENSOARG00000020797 | *TPM1* | 216767,82 | protein_coding |
| ENSOARG00000005466 | *TRIM63* | 213120,51 | protein_coding |
| ENSOARG00000001845 | *TPM3* | 203241,19 | protein_coding |
| ENSOARG00000017679 |  | 199337,47 | protein_coding |
| ENSOARG00000020482 | *PDE4DIP* | 191719,01 | protein_coding |
| ENSOARG00000000016 | *MT-CO1* | 190272,54 | protein_coding |
| ENSOARG00000007876 | *MYOZ1* | 185557,41 | protein_coding |
| ENSOARG00000019190 | *MYL1* | 185209,87 | protein_coding |
| ENSOARG00000020919 | *YBX3* | 182897,13 | protein_coding |
| ENSOARG00000006566 | *TNNC2* | 182510,32 | protein_coding |
| ENSOARG00000011109 | *EEF2* | 175773,68 | protein_coding |
| ENSOARG00000007924 | *PDLIM3* | 173610,63 | protein_coding |
| ENSOARG00000005438 | *OBSCN* | 167581,66 | protein_coding |
| ENSOARG00000005506 | *ENO3* | 165795,80 | protein_coding |
| ENSOARG00000020658 | *TXNIP* | 164811,72 | protein_coding |
| ENSOARG00000013964 | *MYBPC2* | 151950,52 | protein_coding |
| ENSOARG00000000033 | *MT-ND6* | 147041,24 | protein_coding |
| ENSOARG00000026131 |  | 131293,02 | lincRNA |
| ENSOARG00000007666 | *SLC25A4* | 123078,03 | protein_coding |
| ENSOARG00000010213 | *MYOM1* | 119738,24 | protein_coding |
| ENSOARG00000007343 | *NFE2L1* | 117127,62 | protein_coding |
| ENSOARG00000008306 | *ATP5B* | 116588,89 | protein_coding |
| ENSOARG00000018207 |  | 113377,30 | protein_coding |
| ENSOARG00000005748 | *MYOM2* | 112467,32 | protein_coding |
| ENSOARG00000016562 | *GNAS* | 111964,17 | protein_coding |
| ENSOARG00000000936 | *LMOD2* | 111166,82 | protein_coding |
| ENSOARG00000000611 | *LDB3* | 109749,99 | protein_coding |
| ENSOARG00000008405 | *CASQ1* | 105083,76 | protein_coding |
| ENSOARG00000010069 | *MYL6* | 103940,37 | protein_coding |
| ENSOARG00000014048 | *CCNG1* | 103829,21 | protein_coding |
| ENSOARG00000009343 | *HSP90AB1* | 102914,05 | protein_coding |
| ENSOARG00000011805 | *RPLP0* | 102533,16 | protein_coding |
| ENSOARG00000019300 | *PFKM* | 102413,86 | protein_coding |
| ENSOARG00000018666 | *RPLP1* | 100960,71 | protein_coding |
| ENSOARG00000013306 | *ALPK3* | 98557,57 | protein_coding |
| ENSOARG00000004431 | *XIRP2* | 96442,69 | protein_coding |
| ENSOARG00000016775 | *ATF4* | 96383,41 | protein_coding |
| ENSOARG00000012740 | *ZFAND5* | 94094,39 | protein_coding |
| ENSOARG00000014988 | *AHNAK* | 89998,75 | protein_coding |
| ENSOARG00000015514 | *MYOT* | 89401,49 | protein_coding |
| ENSOARG00000018755 | *TNNI1* | 88976,14 | protein_coding |
| ENSOARG00000000491 | *SRRM2* | 87829,78 | protein_coding |
| ENSOARG00000018177 | *UBC* | 86687,86 | protein_coding |
| ENSOARG00000004362 | *KIF1C* | 85783,81 | protein_coding |
| ENSOARG00000004399 | *UBB* | 85168,77 | protein_coding |
| ENSOARG00000010995 | *PPDPF* | 84579,65 | protein_coding |
| ENSOARG00000006670 | *ACTN3* | 81868,25 | protein_coding |
| ENSOARG00000018307 | *ACO2* | 80357,30 | protein_coding |
| ENSOARG00000015194 | *CRYAB* | 79607,39 | protein_coding |
| ENSOARG00000020447 | *YBX1* | 78804,86 | protein_coding |
| ENSOARG00000000037 |  | 78761,88 | Mt_tRNA |
| ENSOARG00000006729 | *TACC2* | 77606,62 | protein_coding |
| ENSOARG00000015846 | *PLEC* | 77150,89 | protein_coding |
| ENSOARG00000013191 | *RPS11* | 77011,58 | protein_coding |
| ENSOARG00000014369 | *SVIL* | 76893,02 | protein_coding |
| ENSOARG00000006867 | *MYOM3* | 75575,48 | protein_coding |
| ENSOARG00000020529 | *CAPN3* | 74200,88 | protein_coding |
| ENSOARG00000005473 | *GSN* | 74149,75 | protein_coding |
| ENSOARG00000015142 | *EEF1G* | 70959,64 | protein_coding |
| ENSOARG00000013116 | *CA3* | 70157,12 | protein_coding |
| ENSOARG00000020020 | *TUBA4A* | 69255,29 | protein_coding |
| ENSOARG00000012220 | *GMPR* | 68849,21 | protein_coding |
| ENSOARG00000003903 | *HSP90AA1* | 67756,94 | protein_coding |
| ENSOARG00000018757 | *GLUL* | 67361,24 | protein_coding |
| ENSOARG00000001603 | *FABP3* | 67121,89 | protein_coding |
| ENSOARG00000008913 | *CSRP3* | 65283,41 | protein_coding |
| ENSOARG00000002247 |  | 65114,45 | pseudogene |
| ENSOARG00000013571 | *RPS17* | 62208,90 | protein_coding |
| ENSOARG00000009602 | *LDHA* | 61127,01 | protein_coding |
| ENSOARG00000000683 | *PTP4A2* | 60961,02 | protein_coding |
| ENSOARG00000009032 | *SPARC* | 60598,66 | protein_coding |
| ENSOARG00000003386 |  | 60294,84 | protein_coding |
| ENSOARG00000016476 | *COL3A1* | 60076,24 | protein_coding |
| ENSOARG00000009990 | *SYNM* | 59842,81 | protein_coding |
| ENSOARG00000016717 | *MYOZ2* | 59519,73 | protein_coding |
| ENSOARG00000002910 | *ATP5A1* | 59479,71 | protein_coding |
| ENSOARG00000020060 | *PABPC4* | 59312,24 | protein_coding |
| ENSOARG00000002683 | *SQSTM1* | 58832,80 | protein_coding |
| ENSOARG00000013633 | *PGAM2* | 58746,10 | protein_coding |
| ENSOARG00000000021 | *MT-ATP8* | 58594,93 | protein_coding |
| ENSOARG00000001219 | *TXLNB* | 58122,90 | protein_coding |
| ENSOARG00000019443 |  | 58098,45 | pseudogene |
| ENSOARG00000006957 | *PSAP* | 57994,70 | protein_coding |
| ENSOARG00000012193 | *VCP* | 56791,28 | protein_coding |
| ENSOARG00000010243 | *EIF4G2* | 56641,59 | protein_coding |
| ENSOARG00000017449 | *CACNA1S* | 56569,71 | protein_coding |
| ENSOARG00000000606 | *AC006254.1* | 56101,39 | protein_coding |
| ENSOARG00000001573 | *MYLK2* | 55435,21 | protein_coding |
| ENSOARG00000008276 | *NACA2* | 55106,19 | protein_coding |
| ENSOARG00000013650 | *MDH2* | 54298,48 | protein_coding |
| ENSOARG00000013272 |  | 54176,95 | protein_coding |
| ENSOARG00000020946 | *TMOD4* | 54074,69 | protein_coding |
| ENSOARG00000016430 | *ACACB* | 53159,53 | protein_coding |
| ENSOARG00000009978 | *FBXO32* | 52838,66 | protein_coding |
| ENSOARG00000007513 | *SYNPO* | 52566,71 | protein_coding |
| ENSOARG00000003677 |  | 52137,65 | protein_coding |
| ENSOARG00000002647 | *MAP4* | 51885,71 | protein_coding |
| ENSOARG00000011328 | *VIM* | 51604,86 | protein_coding |
| ENSOARG00000001136 |  | 51494,45 | protein_coding |
| ENSOARG00000014339 |  | 51431,46 | protein_coding |
| ENSOARG00000001947 | *C10orf71* | 50934,23 | protein_coding |
| ENSOARG00000008297 | *ATP1A2* | 50533,34 | protein_coding |
| ENSOARG00000013748 | *HSPB1* | 50423,67 | protein_coding |
| ENSOARG00000018519 |  | 50278,43 | protein_coding |
| ENSOARG00000020596 | *EIF4G1* | 50214,70 | protein_coding |
| ENSOARG00000001499 | *COQ8A* | 49859,75 | protein_coding |
| ENSOARG00000003064 | *DYNC1H1* | 49446,26 | protein_coding |
| ENSOARG00000018593 | *HADHA* | 48438,47 | protein_coding |
| ENSOARG00000010033 | *LMOD3* | 48290,26 | protein_coding |
| ENSOARG00000010582 |  | 48182,07 | protein_coding |
| ENSOARG00000006712 | *KIAA0368* | 47845,65 | protein_coding |
| ENSOARG00000009101 |  | 47743,39 | protein_coding |
| ENSOARG00000018680 | *HADHB* | 47398,81 | protein_coding |
| ENSOARG00000004871 | *COL1A1* | 47368,43 | protein_coding |
| ENSOARG00000015619 |  | 46966,05 | protein_coding |
| ENSOARG00000001508 | *COL1A2* | 46895,66 | protein_coding |
| ENSOARG00000014168 | *ASB2* | 46612,58 | protein_coding |
| ENSOARG00000003474 | *SPTBN1* | 46471,79 | protein_coding |
| ENSOARG00000003848 | *RPS5* | 46308,77 | protein_coding |
| ENSOARG00000020687 | *USP13* | 46156,86 | protein_coding |
| ENSOARG00000000142 | *HSPA8* | 45769,30 | protein_coding |
| ENSOARG00000015094 | *UQCRC2* | 44921,57 | protein_coding |
| ENSOARG00000002814 |  | 44247,98 | protein_coding |
| ENSOARG00000011503 | *COX4I1* | 44223,53 | protein_coding |
| ENSOARG00000016188 | *BIN1* | 43857,46 | protein_coding |
| ENSOARG00000016963 |  | 43680,36 | protein_coding |
| ENSOARG00000012645 | *RPL8* | 43677,39 | protein_coding |
| ENSOARG00000022521 | *NEAT1_3* | 43506,22 | misc_RNA |
| ENSOARG00000012014 |  | 43178,68 | protein_coding |
| ENSOARG00000011282 | *SERBP1* | 43086,80 | protein_coding |
| ENSOARG00000010662 | *ACADVL* | 42974,16 | protein_coding |
| ENSOARG00000015630 | *RAF1* | 42852,63 | protein_coding |
| ENSOARG00000006055 | *SORBS1* | 42712,58 | protein_coding |
| ENSOARG00000012970 | *SLC25A3* | 42339,10 | protein_coding |
| ENSOARG00000012682 | *HRC* | 41901,16 | protein_coding |
| ENSOARG00000018093 | *PPP1R27* | 41868,55 | protein_coding |
| ENSOARG00000017870 | *HDLBP* | 41538,06 | protein_coding |
| ENSOARG00000000286 | *GOLGA4* | 41181,63 | protein_coding |
| ENSOARG00000016537 | *HSPA9* | 41162,36 | protein_coding |
| ENSOARG00000013169 |  | 41113,45 | protein_coding |
| ENSOARG00000007328 | *RPS14* | 40661,43 | protein_coding |
| ENSOARG00000010362 | *MYL12A* | 39810,73 | protein_coding |
| ENSOARG00000018886 | *PKM* | 39701,80 | protein_coding |
| ENSOARG00000011523 | *RPS3* | 39661,05 | protein_coding |
| ENSOARG00000007288 |  | 39520,99 | protein_coding |
| ENSOARG00000004251 |  | 39443,93 | protein_coding |
| ENSOARG00000003782 | *B2M* | 39431,33 | protein_coding |
| ENSOARG00000020715 | *RMND5A* | 39131,21 | protein_coding |
| ENSOARG00000007183 | *BAG6* | 38583,60 | protein_coding |
| ENSOARG00000020600 | *PSMD2* | 38554,70 | protein_coding |
| ENSOARG00000004817 | *SPEG* | 38474,67 | protein_coding |
| ENSOARG00000016759 | *EIF4B* | 38293,12 | protein_coding |
| ENSOARG00000001717 | *GOT2* | 38170,11 | protein_coding |
| ENSOARG00000013747 | *ANKRD23* | 37856,65 | protein_coding |
| ENSOARG00000019120 | *MYO18B* | 37778,85 | protein_coding |
| ENSOARG00000011188 | *MYH7B* | 37633,61 | protein_coding |
| ENSOARG00000013082 | *PFKFB3* | 37269,76 | protein_coding |
| ENSOARG00000002753 | *RPS9* | 37108,96 | protein_coding |
| ENSOARG00000014862 | *OGDH* | 36717,70 | protein_coding |
| ENSOARG00000007188 | *RAD23B* | 36439,08 | protein_coding |
| ENSOARG00000008072 | *TRDN* | 36399,80 | protein_coding |
| ENSOARG00000020231 | *RPS3A* | 36364,97 | protein_coding |
| ENSOARG00000006115 | *COL4A1* | 36341,26 | protein_coding |

**Supplementary Table S3. Expression levels in the skeletal muscle of Appenninica × Sarda lambs^1^ of 13 genes that are highly expressed in the longissimus dorsi muscle of five Spanish ovine breeds.**

^1^Reference: Sabino et al., (*BMC Genomics*, 2018 Apr 4;19(1):236).

^2^ Gene Expression Omnibus (GEO) accession number.

^3^Ranking of genes according to their expression levels (ranking = 1 corresponds to the most expressed gene).

| **Gene** | **Sabino et al., (2018)**^1^**.** | | | | | | **Our study** | | | | | | | | | |
| --- | --- | --- | --- | --- | --- | --- | --- | --- | --- | --- | --- | --- | --- | --- | --- | --- |
|  |  |  |  |  |  |  |  |  |  |  |  |  |  |  |  |  |
|  | **GSM2997699**^2^ | | **GSM2997701**^2^ | | **GSM2997702**^2^ | | **Ripollesa** | | **Gallega** | | **Xisqueta** | | **Canaria de Pelo** | | **Roja Mallorquina** | |
|  | Normalized counts | Ranking^3^ | Normalized counts | Ranking^3^ | Normalized counts | Ranking^3^ | Normalized counts | Ranking^3^ | Normalized counts | Ranking^3^ | Normalized counts | Ranking^3^ | Normalized counts | Ranking^3^ | Normalized counts | Ranking^3^ |
| *ACTA1* | 503624.7 | 2 | 408336.4 | 2 | 522132.1 | 2 | 7214440.3 | 1 | 12126628.5 | 1 | 4548577.3 | 1 | 2063336.2 | 1 | 2019923.8 | 1 |
| *ALDOA* | 299117.9 | 5 | 180023.9 | 8 | 219144.9 | 9 | 2569599.7 | 3 | 4183403.5 | 3 | 1720349.4 | 17 | 385197.2 | 23 | 400262.4 | 7 |
| *ATP2A1* | 163974.2 | 16 | 116760.1 | 16 | 111805.2 | 19 | 1512547.4 | 9 | 2594051.6 | 9 | 1024738.3 | 21 | 544089.6 | 13 | 338979.0 | 13 |
| *CKM* | 291980.1 | 6 | 332901.1 | 3 | 367118.6 | 3 | 5094719.7 | 2 | 8709202.2 | 2 | 3069686.0 | 8 | 1274471.8 | 4 | 1168356.0 | 3 |
| *GAPDH* | 245919.1 | 9 | 142760.9 | 12 | 160965.5 | 13 | 2542389.1 | 4 | 4164298.1 | 4 | 1567346.6 | 19 | 474381.6 | 15 | 386182.2 | 9 |
| *MB* | 241415.6 | 10 | 157399.0 | 11 | 222453.5 | 8 | 1028787.4 | 15 | 2922463.6 | 8 | 907932.3 | 6 | 628754.5 | 10 | 1204940.0 | 16 |
| *MT-CYB* | 398983.1 | 3 | 299653.3 | 4 | 172235.7 | 11 | 792195.4 | 20 | 1089604.5 | 21 | 2091788.8 | 3 | 1088718.8 | 6 | 1555377.5 | 5 |
| *MYH2* | 160771.2 | 17 | 137648.7 | 14 | 106780.1 | 20 | 1077205.4 | 13 | 1414278.8 | 17 | 2210832.8 | 2 | 1041219.9 | 7 | 1620385.5 | 4 |
| *MYH7* | 221978.2 | 12 | 226757.6 | 5 | 190062.7 | 10 | 1136271.0 | 12 | 3270982.7 | 6 | 1566826.4 | 4 | 1530530.9 | 3 | 1334705.2 | 10 |
| *MYLPF* | 269164.3 | 8 | 139778.2 | 13 | 271045.4 | 5 | 2111536.2 | 6 | 3224258.7 | 7 | 1613947.5 | 13 | 412028.9 | 21 | 552926.3 | 8 |
| *RYR1* | 44584.3 | 51 | 45692.8 | 35 | 29619.1 | 55 | 864342.4 | 19 | 1203450.5 | 18 | 623739.9 | 28 | 417222.8 | 20 | 253160.8 | 22 |
| *TPM2* | 226317.2 | 11 | 159773.3 | 10 | 302778.4 | 4 | 1594570.9 | 8 | 3355183.9 | 5 | 1044548.1 | 9 | 1141913.1 | 5 | 1059813.7 | 12 |
| *TTN* | 328538.1 | 4 | 209409.2 | 6 | 36223.8 | 47 | 977146.0 | 17 | 589510.6 | 34 | 1869300.4 | 7 | 1623023.9 | 2 | 1169949.9 | 6 |

**Supplementary Table S4. Pathways identified by ReactomeFIViz as enriched in the set of 200 top expressed genes in the longissimus dorsi muscle of five sheep breeds.**

| **RIPOLLESA** | | | | |  |
| --- | --- | --- | --- | --- | --- |
|  |  |  |  |  |  |
| **GeneSet** | | ***P*-value** | **FDR** | **Nodes** |  |
| Striated Muscle Contraction(R) | | 1.11E-16 | 1.85E-14 | *ACTN3, TPM3, ACTN2, TPM2, TPM1, MYL1, MYL2, MYL3, NEB, MYBPC1, MYBPC2, TNNC1, TNNC2, MYH3, TNNT1, TTN, TNNI1, TNNI2, DES, TCAP, VIM* |  |
| The citric acid (TCA) cycle and respiratory electron transport(R) | | 1.11E-16 | 1.85E-14 | *MT-ND6, MT-ND5, MT-CO1, UQCRC1, UQCRC2, ATP5B, LDHA, ATP5D, CYC1, NDUFV1, ACO2, MT-CYB, COX4I1, ATP5G3, ATP5G1, MT-ATP6, UQCRFS1, NDUFS2, ATP5A1, COX5B, MDH2* |  |
| Cardiac muscle contraction(K) | | 1.20E-14 | 1.33E-12 | *TPM3, TPM2, TPM1, MYL2, MYL3, UQCRC1, UQCRC2, CYC1, COX4I1, ATP2A2, UQCRFS1, TNNC1, MYH7, COX5B, ACTC1* |  |
| Parkinson's disease(K) | | 4.57E-12 | 3.79E-10 | *UQCRC1, VDAC2, UQCRC2, ATP5B, UBB, ATP5D, CYC1, NDUFV1, COX4I1, ATP5G3, ATP5G1, UQCRFS1, NDUFS2, SLC25A4, ATP5A1, COX5B* |  |
| Alzheimer's disease(K) | | 5.35E-11 | 3.53E-09 | *UQCRC1, UQCRC2, ATP5B, ATP5D, CYC1, NDUFV1, COX4I1, ATP2A2, ATP2A1, ATP5G3, ATP5G1, UQCRFS1, NDUFS2, GAPDH, ATP5A1, COX5B* |  |
| Hypertrophic cardiomyopathy (HCM)(K) | | 1.56E-10 | 8.56E-09 | *ACTG1, TPM3, TPM2, TPM1, MYL2, MYL3, ATP2A2, TNNC1, MYH7, TTN, DES, ACTC1* |  |
| Eukaryotic Translation Elongation(R) | | 2.64E-10 | 1.24E-08 | *RPL8, RPS14, RPS17, RPS11, RPS5, RPL29, EEF2, RPL3L, RPLP1, RPLP0, EEF1G, RPS3* |  |
| Dilated cardiomyopathy(K) | | 3.41E-10 | 1.40E-08 | *ACTG1, TPM3, TPM2, TPM1, MYL2, MYL3, ATP2A2, TNNC1, MYH7, TTN, DES, ACTC1* |  |
| Oxidative phosphorylation(K) | | 2.83E-09 | 1.05E-07 | *UQCRC1, UQCRC2, ATP5B, ATP5D, CYC1, NDUFV1, COX4I1, ATP5G3, ATP5G1, UQCRFS1, NDUFS2, ATP5A1, COX5B* |  |
| Huntington's disease(K) | | 3.44E-09 | 1.14E-07 | *UQCRC1, VDAC2, UQCRC2, ATP5B, ATP5D, CYC1, NDUFV1, COX4I1, ATP5G3, ATP5G1, UQCRFS1, NDUFS2, SLC25A4, ATP5A1, COX5B* |  |
| Glycolysis / Gluconeogenesis(K) | | 4.17E-09 | 1.25E-07 | *ENO3, LDHA, PGK1, GPI, PGM1, TPI1, PGAM2, PKM, ALDOA, GAPDH* |  |
| Nonsense-Mediated Decay (NMD)(R) | | 2.75E-08 | 7.42E-07 | *RPL8, RPS14, RPS17, RPS11, RPS5, RPL29, RPL3L, EIF4G1, RPLP1, RPLP0, RPS3* |  |
| Eukaryotic Translation Termination(R) | | 3.43E-08 | 8.57E-07 | *RPL8, RPS14, RPS17, RPS11, RPS5, RPL29, RPL3L, RPLP1, RPLP0, RPS3* |  |
| Carbon metabolism(K) | | 5.21E-08 | 1.20E-06 | *ENO3, PGK1, GOT2, ACO2, GPI, TPI1, PGAM2, PKM, ALDOA, GAPDH, MDH2* |  |
| Eukaryotic Translation Initiation(R) | | 5.69E-08 | 1.25E-06 | *RPL8, RPS14, RPS17, RPS11, RPS5, RPL29, RPL3L, EIF4G1, RPLP1, RPLP0, RPS3* |  |
| Biosynthesis of amino acids(K) | | 1.43E-07 | 2.86E-06 | *ENO3, PGK1, GOT2, ACO2, TPI1, PGAM2, PKM, ALDOA, GAPDH* |  |
| Nicotinic acetylcholine receptor signaling pathway(P) | | 1.86E-07 | 3.54E-06 | *ACTG1, ACTA1, MYH2, MYH3, MYH7, ACTC1* |  |
| SRP-dependent cotranslational protein targeting to membrane(R) | | 2.64E-07 | 4.76E-06 | *RPL8, RPS14, RPS17, RPS11, RPS5, RPL29, RPL3L, RPLP1, RPLP0, RPS3* |  |
| Selenoamino acid metabolism(R) | | 2.88E-07 | 4.90E-06 | *RPL8, RPS14, RPS17, RPS11, RPS5, RPL29, RPL3L, RPLP1, RPLP0, RPS3* |  |
| Metabolism of carbohydrates(R) | | 3.69E-07 | 5.90E-06 | *ENO3, UBB, PGK1, GOT2, GPI, PYGM, GYS1, PGM1, TPI1, PGAM2, PKM, ALDOA, GAPDH, MDH2* |  |
| Ribosome(K) | | 2.84E-06 | 4.25E-05 | *RPL8, RPS14, RPS17, RPS11, RPS5, RPL29, RPL3L, RPLP1, RPLP0, RPS3* |  |
| Focal adhesion(K) | | 2.96E-06 | 4.43E-05 | *MYLK2, ACTG1, ACTN3, ACTN2, COL4A2, COL4A1, MYL2, MYLPF, COL1A1, COL1A2, FLNC, COL3A1* |  |
| Adrenergic signaling in cardiomyocytes(K) | | 5.56E-06 | 7.78E-05 | *TPM3, TPM2, TPM1, MYL2, MYL3, ATF4, ATP2A2, TNNC1, MYH7, ACTC1* |  |
| Amoebiasis(K) | | 2.63E-05 | 3.41E-04 | *ACTN3, ACTN2, COL4A2, COL4A1, COL1A1, COL1A2, HSPB1, COL3A1* |  |
| Smooth Muscle Contraction(R) | | 3.40E-05 | 4.41E-04 | *TPM3, TPM2, TPM1, MYL6, MYLPF* |  |
| Metabolic pathways(K) | | 4.15E-05 | 4.98E-04 | *ENO3, UQCRC1, UQCRC2, ATP5B, LDHA, ATP5D, PGK1, CYC1, NDUFV1, GOT2, ACO2, GPI, COX4I1, PYGM, ATP5G3, ATP5G1, UQCRFS1, PGM1, TPI1, PGAM2, PKM, NDUFS2, ALDOA, GAPDH, ATP5A1, COX5B, CKMT2, CKM, MDH2* |  |
| Non-alcoholic fatty liver disease (NAFLD)(K) | | 4.39E-05 | 5.27E-04 | *UQCRC1, UQCRC2, ATF4, CYC1, NDUFV1, COX4I1, UQCRFS1, NDUFS2, COX5B* |  |
| Tight junction(K) | | 1.43E-04 | 1.57E-03 | *ACTG1, ACTN3, ACTN2, MYL2, MYLPF, MYH2, MYH3, MYH7* |  |
| Integrin signalling pathway(P) | | 3.53E-04 | 3.88E-03 | *ACTG1, ACTN3, ACTN2, COL4A2, COL4A1, COL1A1, COL1A2, COL3A1* |  |
| Huntington disease(P) | | 3.80E-04 | 4.18E-03 | *ACTG1, CAPN3, CYC1, ACTA1, GAPDH, DYNC1H1, ACTC1* |  |
| Viral myocarditis(K) | | 4.60E-04 | 4.60E-03 | *ACTG1, HLA-E, EIF4G2, EIF4G1, MYH7* |  |
| Calcium signaling pathway(K) | | 7.94E-04 | 7.87E-03 | *MYLK2, VDAC2, RYR1, ATP2A2, ATP2A1, TNNC1, TNNC2, SLC25A4* |  |
| HIF-1-alpha transcription factor network(N) | | 8.19E-04 | 7.87E-03 | *LDHA, PGK1, PGM1, PKM, ALDOA* |  |
| Glucagon signaling pathway(K) | | 8.74E-04 | 7.87E-03 | *ATF4, LDHA, PYGM, GYS1, PGAM2, PKM* |  |
| downregulated of mta-3 in er-negative breast tumors(B) | | 1.05E-03 | 9.48E-03 | *TUBA4A, HSPB1, ALDOA* |  |
| Arrhythmogenic right ventricular cardiomyopathy (ARVC)(K) | | 1.36E-03 | 0.0122 | *ACTG1, ACTN3, ACTN2, ATP2A2, DES* |  |
| Antigen processing and presentation(K) | | 1.61E-03 | 0.0136 | *B2M, HLA-E, HSPA8, HSP90AB1, HSP90AA1* |  |
| Cellular response to heat stress(R) | | 1.70E-03 | 0.0136 | *VCP, HSPA8, HSP90AB1, HSP90AA1, CRYAB* |  |
| Response to elevated platelet cytosolic Ca2+(R) | | 2.23E-03 | 0.0178 | *SPARC, ACTN2, TUBA4A, ALDOA, TTN* |  |
| cGMP-PKG signaling pathway(K) | | 2.42E-03 | 0.0193 | *MYLK2, VDAC2, ATF4, ATP2A2, ATP2A1, SLC25A4, MYH7* |  |
| ECM-receptor interaction(K) | | 2.72E-03 | 0.0218 | *COL4A2, COL4A1, COL1A1, COL1A2, COL3A1* |  |
| Protein digestion and absorption(K) | | 3.15E-03 | 0.022 | *COL4A2, COL4A1, COL1A1, COL1A2, COL3A1* |  |
| Starch and sucrose metabolism(K) | | 3.40E-03 | 0.0238 | *GPI, PYGM, GYS1, PGM1* |  |
| Pentose phosphate pathway(K) | | 4.04E-03 | 0.0283 | *GPI, PGM1, ALDOA* |  |
| Binding and Uptake of Ligands by Scavenger Receptors(R) | | 5.45E-03 | 0.0381 | *SPARC, COL4A2, COL4A1, COL1A1, COL1A2, HSP90AA1, COL3A1* |  |
| HIF-1 signaling pathway(K) | | 5.53E-03 | 0.0387 | *ENO3, LDHA, PGK1, ALDOA, GAPDH* |  |
| Beta1 integrin cell surface interactions(N) | | 6.04E-03 | 0.0393 | *COL4A1, COL1A1, COL1A2, COL3A1* |  |
| regulators of bone mineralization(B) | | 6.55E-03 | 0.0393 | *COL4A2, COL4A1* |  |
| Regulation of actin cytoskeleton(K) | | 9.09E-03 | 0.0545 | *MYLK2, ACTG1, ACTN3, ACTN2, MYL2, MYLPF, GSN* |  |
| Validated targets of C-MYC transcriptional activation(N) | | 9.35E-03 | 0.0561 | *LDHA, EIF4G1, HSP90AA1, GAPDH* |  |
| Leukocyte transendothelial migration(K) | | 9.62E-03 | 0.0577 | *ACTG1, ACTN3, ACTN2, MYL2, MYLPF* |  |
| Pyruvate metabolism(K) | | 9.73E-03 | 0.0584 | *LDHA, PKM, MDH2* |  |
| stress induction of hsp regulation(B) | | 0.0104 | 0.0623 | *HSPB1, ACTA1* |  |
| Protein processing in endoplasmic reticulum(K) | | 0.0106 | 0.0628 | *ATF4, VCP, HSPA8, HSP90AB1, HSP90AA1, CRYAB* |  |
| Beta3 integrin cell surface interactions(N) | | 0.0118 | 0.0628 | *COL4A1, COL1A1, COL1A2* |  |
| Cysteine and methionine metabolism(K) | | 0.0126 | 0.0628 | *LDHA, GOT2, MDH2* |  |
| PI3K-Akt signaling pathway(K) | | 0.0132 | 0.0659 | *COL4A2, COL4A1, ATF4, COL1A1, COL1A2, HSP90AB1, GYS1, HSP90AA1, COL3A1* |  |
| Platelet activation(K) | | 0.0141 | 0.0705 | *MYLK2, ACTG1, COL1A1, COL1A2, COL3A1* |  |
| Regulation of mRNA stability by proteins that bind AU-rich elements(R) | | 0.0148 | 0.0739 | *UBB, HSPA8, EIF4G1, HSPB1* |  |
| Apoptotic execution phase(R) | | 0.015 | 0.0748 | *PLEC, GSN, VIM* |  |
| 2-Oxocarboxylic acid metabolism(K) | | 0.015 | 0.075 | *GOT2, ACO2* |  |
| Arginine and proline metabolism(K) | | 0.0176 | 0.0879 | *GOT2, CKMT2, CKM* |  |
| Extracellular matrix organization(R) | | 0.019 | 0.0948 | *SPARC, COL4A2, COL4A1, COL1A1, COL1A2, PLEC, COL3A1* |  |
| Caspase cascade in apoptosis(N) | | 0.0195 | 0.097 | *ACTA1, GSN, VIM* |  |
| Legionellosis(K) | | 0.0225 | 0.097 | *VCP, HSPA8, EEF1G* |  |
| Estrogen signaling pathway(K) | | 0.0233 | 0.097 | *ATF4, HSPA8, HSP90AB1, HSP90AA1* |  |
| intrinsic prothrombin activation pathway(B) | | 0.0243 | 0.097 | *COL4A2, COL4A1* |  |
| Nephrin interactions(R) | | 0.0243 | 0.097 | *ACTN3, ACTN2* |  |
| Viral carcinogenesis(K) | | 0.0249 | 0.0996 | *ACTN3, ACTN2, HLA-E, ATF4, PKM, GSN* |  |
| Oxytocin signaling pathway(K) | | 0.0296 | 0.1183 | *MYLK2, ACTG1, MYL6, EEF2, RYR1* |  |
| Cardiac conduction(R) | | 0.0299 | 0.1194 | *SLN, RYR1, ATP2A2, ATP2A1* |  |
| VEGFR3 signaling in lymphatic endothelium(N) | | 0.0307 | 0.1227 | *COL1A1, COL1A2* |  |
| Cytoskeletal regulation by Rho GTPase(P) | | 0.0321 | 0.1283 | *MYLK2* |  |
| transcriptional activation of dbpb from mrna(B) | | 0.0321 | 0.1283 | *YBX1* |  |
| Mitotic G2-G2/M phases(R) | | 0.0335 | 0.1339 | *TUBA4A, UBB, HSP90AA1, DYNC1H1* |  |
| Semaphorin interactions(R) | | 0.0344 | 0.1377 | *MYL6, HSP90AB1, HSP90AA1* |  |
| ISG15 antiviral mechanism(R) | | 0.0371 | 0.1436 | *UBB, EIF4G2, EIF4G1* |  |
| Central carbon metabolism in cancer(K) | | 0.0371 | 0.1436 | *LDHA, PGAM2, PKM* |  |
| Glyoxylate and dicarboxylate metabolism(K) | | 0.0377 | 0.1436 | *ACO2, MDH2* |  |
| Ion channel transport(R) | | 0.0402 | 0.1436 | *SLN, UBB, RYR1, ATP2A2, ATP2A1* |  |
| Pyruvate metabolism(P) | | 0.0426 | 0.1436 | *PKM* |  |
| Citrate cycle (TCA cycle)(K) | | 0.0427 | 0.1436 | *ACO2, MDH2* |  |
| Adherens junction(K) | | 0.0459 | 0.1436 | *ACTG1, ACTN3, ACTN2* |  |
| Fructose and mannose metabolism(K) | | 0.0479 | 0.1436 | *TPI1, ALDOA* |  |
| N-cadherin signaling events(N) | | 0.0506 | 0.1517 | *MYL2, GSN* |  |
| FAS signaling pathway(P) | | 0.0506 | 0.1517 | *CYC1, GSN* |  |
| Glycolysis(P) | | 0.0529 | 0.1587 | *PGK1* |  |
| antigen processing and presentation(B) | | 0.0529 | 0.1587 | *B2M* |  |
| Phenylalanine. tyrosine and tryptophan biosynthesis(K) | | 0.0529 | 0.1587 | *GOT2* |  |
| nfat and hypertrophy of the heart (B) | | 0.0589 | 0.1768 | *MYL2, ACTA1* |  |
| C-MYB transcription factor network(N) | | 0.0589 | 0.1768 | *HSPA8, COL1A2, SLC25A3* |  |
| Validated transcriptional targets of AP1 family members Fra1 and Fra2(N) | | 0.0618 | 0.1855 | *ATF4, COL1A2* |  |
| cell to cell adhesion signaling(B) | | 0.0631 | 0.1894 | *ACTA1* |  |
| Pre-NOTCH Expression and Processing(R) | | 0.0648 | 0.1943 | *ATP2A2, ATP2A1* |  |
| Salmonella infection(K) | | 0.0679 | 0.2037 | *ACTG1, FLNC, DYNC1H1* |  |
| Aurora B signaling(N) | | 0.0708 | 0.2124 | *DES, VIM* |  |
| Prostate cancer(K) | | 0.0736 | 0.2207 | *ATF4, HSP90AB1, HSP90AA1* |  |
| EPH-Ephrin signaling(R) | | 0.0736 | 0.2207 | *ACTG1, MYL6, HSP90AA1* |  |
| Amyloid fiber formation(R) | | 0.0739 | 0.2216 | *B2M, GSN* |  |
| Class I MHC mediated antigen processing & presentation(R) | | 0.0775 | 0.2324 | *B2M, HLA-E, UBB* |  |
| **GALLEGA** | | | | |  |
|  |  |  |  |  |  |
| **GeneSet** | ***P*-value** | | **FDR** | **Nodes** | |
| Striated Muscle Contraction(R) | 1.11E-16 | | 3.67E-14 | ACTN3, TPM3, ACTN2, TPM2, TPM1, MYL1, MYL2, NEB, MYBPC1, MYBPC2, TNNC1, TNNC2, TNNT1, TTN, TNNI1, TNNI2, DES, TCAP, VIM | |
| The citric acid (TCA) cycle and respiratory electron transport(R) | 3.33E-16 | | 5.50E-14 | MT-ND5, MT-CO1, UQCRC1, UQCRC2, ATP5B, LDHA, ATP5D, CYC1, NDUFV1, ACO2, MT-CYB, COX4I1, ATP5G1, MT-ATP6, UQCRFS1, NDUFS7, NDUFS2, ATP5A1, COX5B, MDH2 | |
| Eukaryotic Translation Elongation(R) | 1.33E-15 | | 1.47E-13 | RPL32, RPL8, RPS14, RPS17, RPS11, RPS9, RPS5, RPL29, EEF2, RPL3L, RPLP1, RPLP0, EEF1G, EEF1D, RPS3, RPL18 | |
| Parkinson's disease(K) | 2.20E-12 | | 1.63E-10 | UQCRC1, VDAC2, UQCRC2, ATP5B, UBB, ATP5D, CYC1, NDUFV1, COX4I1, ATP5G1, UQCRFS1, NDUFS7, NDUFS2, SLC25A4, ATP5A1, COX5B | |
| Cardiac muscle contraction(K) | 2.47E-12 | | 1.63E-10 | TPM3, TPM2, TPM1, MYL2, UQCRC1, UQCRC2, CYC1, COX4I1, ATP2A2, UQCRFS1, TNNC1, MYH7, COX5B | |
| Eukaryotic Translation Termination(R) | 6.16E-12 | | 3.39E-10 | RPL32, RPL8, RPS14, RPS17, RPS11, RPS9, RPS5, RPL29, RPL3L, RPLP1, RPLP0, RPS3, RPL18 | |
| Nonsense-Mediated Decay (NMD)(R) | 7.28E-12 | | 3.42E-10 | RPL32, RPL8, RPS14, RPS17, RPS11, RPS9, RPS5, RPL29, RPL3L, EIF4G1, RPLP1, RPLP0, RPS3, RPL18 | |
| Eukaryotic Translation Initiation(R) | 1.88E-11 | | 7.73E-10 | RPL32, RPL8, RPS14, RPS17, RPS11, RPS9, RPS5, RPL29, RPL3L, EIF4G1, RPLP1, RPLP0, RPS3, RPL18 | |
| Alzheimer's disease(K) | 2.61E-11 | | 9.40E-10 | UQCRC1, UQCRC2, ATP5B, ATP5D, CYC1, NDUFV1, COX4I1, ATP2A2, ATP2A1, ATP5G1, UQCRFS1, NDUFS7, NDUFS2, GAPDH, ATP5A1, COX5B | |
| SRP-dependent cotranslational protein targeting to membrane(R) | 9.37E-11 | | 3.09E-09 | RPL32, RPL8, RPS14, RPS17, RPS11, RPS9, RPS5, RPL29, RPL3L, RPLP1, RPLP0, RPS3, RPL18 | |
| Selenoamino acid metabolism(R) | 1.05E-10 | | 3.15E-09 | RPL32, RPL8, RPS14, RPS17, RPS11, RPS9, RPS5, RPL29, RPL3L, RPLP1, RPLP0, RPS3, RPL18 | |
| Oxidative phosphorylation(K) | 1.60E-09 | | 4.31E-08 | UQCRC1, UQCRC2, ATP5B, ATP5D, CYC1, NDUFV1, COX4I1, ATP5G1, UQCRFS1, NDUFS7, NDUFS2, ATP5A1, COX5B | |
| Huntington's disease(K) | 1.79E-09 | | 4.49E-08 | UQCRC1, VDAC2, UQCRC2, ATP5B, ATP5D, CYC1, NDUFV1, COX4I1, ATP5G1, UQCRFS1, NDUFS7, NDUFS2, SLC25A4, ATP5A1, COX5B | |
| Ribosome(K) | 2.27E-09 | | 5.21E-08 | RPL32, RPL8, RPS14, RPS17, RPS11, RPS9, RPS5, RPL29, RPL3L, RPLP1, RPLP0, RPS3, RPL18 | |
| Glycolysis / Gluconeogenesis(K) | 2.65E-09 | | 5.83E-08 | ENO3, LDHA, PGK1, GPI, PGM1, TPI1, PGAM2, PKM, ALDOA, GAPDH | |
| Metabolism of carbohydrates(R) | 3.00E-08 | | 6.01E-07 | ENO3, HSPG2, UBB, PGK1, GOT2, GPI, PYGM, GYS1, PGM1, TPI1, PGAM2, PKM, ALDOA, GAPDH, MDH2 | |
| Carbon metabolism(K) | 3.22E-08 | | 6.12E-07 | ENO3, PGK1, GOT2, ACO2, GPI, TPI1, PGAM2, PKM, ALDOA, GAPDH, MDH2 | |
| Biosynthesis of amino acids(K) | 9.60E-08 | | 1.73E-06 | ENO3, PGK1, GOT2, ACO2, TPI1, PGAM2, PKM, ALDOA, GAPDH | |
| Hypertrophic cardiomyopathy (HCM)(K) | 2.50E-07 | | 4.25E-06 | TPM3, TPM2, TPM1, MYL2, ATP2A2, TNNC1, MYH7, TTN, DES | |
| Dilated cardiomyopathy(K) | 4.45E-07 | | 7.12E-06 | TPM3, TPM2, TPM1, MYL2, ATP2A2, TNNC1, MYH7, TTN, DES | |
| Non-alcoholic fatty liver disease (NAFLD)(K) | 4.37E-06 | | 6.55E-05 | UQCRC1, UQCRC2, ATF4, CYC1, NDUFV1, COX4I1, UQCRFS1, NDUFS7, NDUFS2, COX5B | |
| Metabolic pathways(K) | 1.63E-05 | | 2.44E-04 | ENO3, UQCRC1, UQCRC2, ATP5B, LDHA, ATP5D, PGK1, CYC1, NDUFV1, GOT2, ACO2, GPI, COX4I1, PYGM, ATP5G1, UQCRFS1, PGM1, TPI1, PGAM2, PKM, NDUFS7, NDUFS2, ALDOA, GAPDH, ATP5A1, COX5B, CKMT2, CKM, MDH2 | |
| Amoebiasis(K) | 1.88E-05 | | 2.63E-04 | ACTN3, ACTN2, COL4A2, COL4A1, COL1A1, HSPB1, LAMB2, COL3A1 | |
| Smooth Muscle Contraction(R) | 2.72E-05 | | 3.54E-04 | TPM3, TPM2, TPM1, MYL6, MYLPF | |
| Focal adhesion(K) | 6.34E-05 | | 8.25E-04 | MYLK2, ACTN3, ACTN2, COL4A2, COL4A1, MYL2, MYLPF, COL1A1, LAMB2, COL3A1 | |
| Adrenergic signaling in cardiomyocytes(K) | 1.66E-04 | | 2.00E-03 | TPM3, TPM2, TPM1, MYL2, ATF4, ATP2A2, TNNC1, MYH7 | |
| ECM-receptor interaction(K) | 3.15E-04 | | 3.78E-03 | HSPG2, COL4A2, COL4A1, COL1A1, LAMB2, COL3A1 | |
| Calcium signaling pathway(K) | 5.86E-04 | | 6.44E-03 | MYLK2, VDAC2, RYR1, ATP2A2, ATP2A1, TNNC1, TNNC2, SLC25A4 | |
| HIF-1-alpha transcription factor network(N) | 6.65E-04 | | 7.31E-03 | LDHA, PGK1, PGM1, PKM, ALDOA | |
| Glucagon signaling pathway(K) | 6.87E-04 | | 7.55E-03 | ATF4, LDHA, PYGM, GYS1, PGAM2, PKM | |
| downregulated of mta-3 in er-negative breast tumors(B) | 9.22E-04 | | 9.22E-03 | TUBA4A, HSPB1, ALDOA | |
| Antigen processing and presentation(K) | 1.31E-03 | | 0.0131 | B2M, HLA-E, HSPA8, HSP90AB1, HSP90AA1 | |
| Nicotinic acetylcholine receptor signaling pathway(P) | 1.63E-03 | | 0.0163 | ACTA1, MYH2, MYH7 | |
| Response to elevated platelet cytosolic Ca2+(R) | 1.82E-03 | | 0.0164 | SPARC, ACTN2, TUBA4A, ALDOA, TTN | |
| cGMP-PKG signaling pathway(K) | 1.86E-03 | | 0.0168 | MYLK2, VDAC2, ATF4, ATP2A2, ATP2A1, SLC25A4, MYH7 | |
| Starch and sucrose metabolism(K) | 2.88E-03 | | 0.0259 | GPI, PYGM, GYS1, PGM1 | |
| Tight junction(K) | 3.29E-03 | | 0.0263 | ACTN3, ACTN2, MYL2, MYLPF, MYH2, MYH7 | |
| Pentose phosphate pathway(K) | 3.55E-03 | | 0.0284 | GPI, PGM1, ALDOA | |
| Extracellular matrix organization(R) | 4.44E-03 | | 0.0355 | SPARC, HSPG2, COL4A2, COL4A1, COL1A1, PLEC, LAMB2, COL3A1 | |
| HIF-1 signaling pathway(K) | 4.56E-03 | | 0.0365 | ENO3, LDHA, PGK1, ALDOA, GAPDH | |
| Beta1 integrin cell surface interactions(N) | 5.14E-03 | | 0.0411 | COL4A1, COL1A1, LAMB2, COL3A1 | |
| regulators of bone mineralization(B) | 5.99E-03 | | 0.042 | COL4A2, COL4A1 | |
| Integrin signalling pathway(P) | 6.28E-03 | | 0.044 | ACTN3, ACTN2, COL4A2, COL4A1, COL1A1, COL3A1 | |
| **ROJA MALLORQUINA** | | | | |  |
|  |  |  |  |  |  |
| **GeneSet** | | ***P*-value** | **FDR** | **Nodes** |  |
| Striated Muscle Contraction(R) | | 1.11E-16 | 1.78E-14 | *ACTN3, TPM3, ACTN2, TPM2, TPM1, MYL1, MYL2, NEB, MYBPC1, MYBPC2, TNNC1, TNNC2, TNNT1, TTN, TNNI1, TNNI2, DES, TCAP, VIM* |  |
| The citric acid (TCA) cycle and respiratory electron transport(R) | | 1.11E-16 | 1.78E-14 | *MT-ND6, MT-ND5, MT-CO1, COX6B1, UQCRC1, UQCRC2, ATP5B, LDHA, CYC1, NDUFV1, ACO2, MT-CYB, COX4I1, ATP5G3, ATP5G1, MT-ATP6, UQCRFS1, NDUFS2, NDUFS1, ATP5A1, COX5B, MDH2, MT-ATP8* |  |
| Parkinson's disease(K) | | 9.55E-15 | 1.02E-12 | *COX6B1, UQCRC1, VDAC2, VDAC1, UQCRC2, ATP5B, UBB, CYC1, NDUFV1, COX4I1, ATP5G3, ATP5G1, UQCRFS1, NDUFS2, NDUFS1, SLC25A4, ATP5A1, COX5B* |  |
| Cardiac muscle contraction(K) | | 1.10E-13 | 8.80E-12 | *TPM3, TPM2, TPM1, COX6B1, MYL2, UQCRC1, UQCRC2, CYC1, COX4I1, ATP2A2, UQCRFS1, TNNC1, MYH7, COX5B* |  |
| Alzheimer's disease(K) | | 1.96E-12 | 1.25E-10 | *COX6B1, UQCRC1, UQCRC2, ATP5B, CYC1, NDUFV1, COX4I1, ATP2A2, ATP2A1, ATP5G3, ATP5G1, UQCRFS1, NDUFS2, NDUFS1, GAPDH, ATP5A1, COX5B* |  |
| Huntington's disease(K) | | 1.69E-11 | 8.94E-10 | *COX6B1, UQCRC1, VDAC2, VDAC1, UQCRC2, ATP5B, CYC1, NDUFV1, COX4I1, ATP5G3, ATP5G1, UQCRFS1, NDUFS2, NDUFS1, SLC25A4, ATP5A1, COX5B* |  |
| Oxidative phosphorylation(K) | | 1.22E-10 | 5.49E-09 | *COX6B1, UQCRC1, UQCRC2, ATP5B, CYC1, NDUFV1, COX4I1, ATP5G3, ATP5G1, UQCRFS1, NDUFS2, NDUFS1, ATP5A1, COX5B* |  |
| Eukaryotic Translation Elongation(R) | | 2.05E-09 | 8.19E-08 | *RPL8, RPS14, RPS17, RPS11, RPS5, EEF2, RPLP1, RPLP0, RPS3A, EEF1G, RPS3* |  |
| Glycolysis / Gluconeogenesis(K) | | 2.42E-09 | 8.46E-08 | *ENO3, LDHA, PGK1, GPI, PGM1, TPI1, PGAM2, PKM, ALDOA, GAPDH* |  |
| Carbon metabolism(K) | | 2.92E-08 | 9.33E-07 | *ENO3, PGK1, GOT2, ACO2, GPI, TPI1, PGAM2, PKM, ALDOA, GAPDH, MDH2* |  |
| Biosynthesis of amino acids(K) | | 8.84E-08 | 2.56E-06 | *ENO3, PGK1, GOT2, ACO2, TPI1, PGAM2, PKM, ALDOA, GAPDH* |  |
| Nonsense-Mediated Decay (NMD)(R) | | 1.71E-07 | 4.44E-06 | *RPL8, RPS14, RPS17, RPS11, RPS5, EIF4G1, RPLP1, RPLP0, RPS3A, RPS3* |  |
| Hypertrophic cardiomyopathy (HCM)(K) | | 2.30E-07 | 5.53E-06 | *TPM3, TPM2, TPM1, MYL2, ATP2A2, TNNC1, MYH7, TTN, DES* |  |
| Eukaryotic Translation Termination(R) | | 2.54E-07 | 5.59E-06 | *RPL8, RPS14, RPS17, RPS11, RPS5, RPLP1, RPLP0, RPS3A, RPS3* |  |
| Eukaryotic Translation Initiation(R) | | 3.30E-07 | 6.93E-06 | *RPL8, RPS14, RPS17, RPS11, RPS5, EIF4G1, RPLP1, RPLP0, RPS3A, RPS3* |  |
| Dilated cardiomyopathy(K) | | 4.10E-07 | 8.20E-06 | *TPM3, TPM2, TPM1, MYL2, ATP2A2, TNNC1, MYH7, TTN, DES* |  |
| Non-alcoholic fatty liver disease (NAFLD)(K) | | 5.13E-07 | 9.23E-06 | *COX6B1, UQCRC1, UQCRC2, ATF4, CYC1, NDUFV1, COX4I1, UQCRFS1, NDUFS2, NDUFS1, COX5B* |  |
| Metabolism of carbohydrates(R) | | 1.18E-06 | 2.01E-05 | *ENO3, UBB, PGK1, GOT2, GPI, PYGM, PGM1, TPI1, PGAM2, PKM, ALDOA, GAPDH, MDH2* |  |
| Smooth Muscle Contraction(R) | | 1.39E-06 | 2.23E-05 | *TPM3, TPM2, TPM1, MYL6, MYL12A, MYLPF* |  |
| SRP-dependent cotranslational protein targeting to membrane(R) | | 1.59E-06 | 2.54E-05 | *RPL8, RPS14, RPS17, RPS11, RPS5, RPLP1, RPLP0, RPS3A, RPS3* |  |
| Selenoamino acid metabolism(R) | | 1.71E-06 | 2.57E-05 | *RPL8, RPS14, RPS17, RPS11, RPS5, RPLP1, RPLP0, RPS3A, RPS3* |  |
| Metabolic pathways(K) | | 4.54E-06 | 6.36E-05 | *ENO3, COX6B1, UQCRC1, UQCRC2, ATP5B, LDHA, PGK1, CYC1, NDUFV1, GOT2, ACO2, GPI, COX4I1, PYGM, ATP5G3, ATP5G1, UQCRFS1, PGM1, TPI1, PGAM2, PKM, NDUFS2, NDUFS1, ALDOA, GAPDH, ATP5A1, COX5B, CKMT2, CKM, MDH2* |  |
| Ribosome(K) | | 1.33E-05 | 1.72E-04 | *RPL8, RPS14, RPS17, RPS11, RPS5, RPLP1, RPLP0, RPS3A, RPS3* |  |
| Focal adhesion(K) | | 5.85E-05 | 7.61E-04 | *MYLK2, ACTN3, ACTN2, COL4A1, MYL2, MYL12A, MYLPF, COL1A1, COL1A2, COL3A1* |  |
| Nicotinic acetylcholine receptor signaling pathway(P) | | 8.65E-05 | 1.04E-03 | *MYH7B, ACTA1, MYH2, MYH7* |  |
| Tight junction(K) | | 9.68E-05 | 1.14E-03 | *ACTN3, ACTN2, MYL2, MYL12A, MYH7B, MYLPF, MYH2, MYH7* |  |
| Calcium signaling pathway(K) | | 1.03E-04 | 1.14E-03 | *MYLK2, VDAC2, VDAC1, RYR1, ATP2A2, ATP2A1, TNNC1, TNNC2, SLC25A4* |  |
| Adrenergic signaling in cardiomyocytes(K) | | 1.56E-04 | 1.71E-03 | *TPM3, TPM2, TPM1, MYL2, ATF4, ATP2A2, TNNC1, MYH7* |  |
| cGMP-PKG signaling pathway(K) | | 3.49E-04 | 3.84E-03 | *MYLK2, VDAC2, VDAC1, ATF4, ATP2A2, ATP2A1, SLC25A4, MYH7* |  |
| HIF-1-alpha transcription factor network(N) | | 6.37E-04 | 6.37E-03 | *LDHA, PGK1, PGM1, PKM, ALDOA* |  |
| Amoebiasis(K) | | 9.22E-04 | 9.22E-03 | *ACTN3, ACTN2, COL4A1, COL1A1, COL1A2, COL3A1* |  |
| Antigen processing and presentation(K) | | 1.26E-03 | 0.012 | *B2M, HLA-E, HSPA8, HSP90AB1, HSP90AA1* |  |
| Cellular response to heat stress(R) | | 1.33E-03 | 0.012 | *VCP, HSPA8, HSP90AB1, HSP90AA1, CRYAB* |  |
| Response to elevated platelet cytosolic Ca2+(R) | | 1.75E-03 | 0.0157 | *SPARC, ACTN2, TUBA4A, ALDOA, TTN* |  |
| Pentose phosphate pathway(K) | | 3.46E-03 | 0.0311 | *GPI, PGM1, ALDOA* |  |
| Glucagon signaling pathway(K) | | 4.03E-03 | 0.0323 | *ATF4, LDHA, PYGM, PGAM2, PKM* |  |
| HIF-1 signaling pathway(K) | | 4.38E-03 | 0.035 | *ENO3, LDHA, PGK1, ALDOA, GAPDH* |  |
| Beta1 integrin cell surface interactions(N) | | 4.97E-03 | 0.0397 | *COL4A1, COL1A1, COL1A2, COL3A1* |  |
| Integrin signalling pathway(P) | | 6.00E-03 | 0.048 | *ACTN3, ACTN2, COL4A1, COL1A1, COL1A2, COL3A1* |  |
| **CANARIA DE PELO** | | | | |  |
|  |  |  |  |  |  |
| **GeneSet** | | ***P*-value** | **FDR** | **Nodes** |  |
| Striated Muscle Contraction(R) | | 1.11E-16 | 4.85E-14 | *ACTN3, TPM3, ACTN2, TPM2, TPM1, MYL1, MYL2, NEB, MYBPC1, MYBPC2, TNNC1, TNNC2, TNNT1, TTN, TNNI1, TNNI2, DES, TCAP, VIM* |  |
| Eukaryotic Translation Elongation(R) | | 1.37E-10 | 2.99E-08 | *RPL8, RPS14, RPS17, RPS11, RPS9, RPS5, EEF2, RPLP1, RPLP0, RPS3A, EEF1G, RPS3* |  |
| Eukaryotic Translation Initiation(R) | | 2.79E-09 | 4.05E-07 | *RPL8, RPS14, RPS17, RPS11, RPS9, RPS5, EIF4G1, RPLP1, RPLP0, RPS3A, RPS3, EIF4B* |  |
| Cardiac muscle contraction(K) | | 1.01E-08 | 1.01E-06 | *TPM3, TPM2, TPM1, MYL2, UQCRC2, COX4I1, ATP2A2, TNNC1, MYH7, ATP1A2* |  |
| The citric acid (TCA) cycle and respiratory electron transport(R) | | 1.16E-08 | 1.01E-06 | *MT-ND6, MT-ND5, MT-CO1, UQCRC2, ATP5B, LDHA, ACO2, MT-CYB, COX4I1, MT-ATP6, ATP5A1, MDH2, MT-ATP8* |  |
| Nonsense-Mediated Decay (NMD)(R) | | 1.53E-08 | 1.10E-06 | *RPL8, RPS14, RPS17, RPS11, RPS9, RPS5, EIF4G1, RPLP1, RPLP0, RPS3A, RPS3* |  |
| Eukaryotic Translation Termination(R) | | 2.01E-08 | 1.24E-06 | *RPL8, RPS14, RPS17, RPS11, RPS9, RPS5, RPLP1, RPLP0, RPS3A, RPS3* |  |
| Smooth Muscle Contraction(R) | | 6.35E-08 | 3.43E-06 | *TPM3, TPM2, TPM1, MYL6, MYL12A, MYLPF, SORBS1* |  |
| SRP-dependent cotranslational protein targeting to membrane(R) | | 1.57E-07 | 7.34E-06 | *RPL8, RPS14, RPS17, RPS11, RPS9, RPS5, RPLP1, RPLP0, RPS3A, RPS3* |  |
| Selenoamino acid metabolism(R) | | 1.71E-07 | 7.34E-06 | *RPL8, RPS14, RPS17, RPS11, RPS9, RPS5, RPLP1, RPLP0, RPS3A, RPS3* |  |
| Hypertrophic cardiomyopathy (HCM)(K) | | 2.30E-07 | 8.98E-06 | *TPM3, TPM2, TPM1, MYL2, ATP2A2, TNNC1, MYH7, TTN, DES* |  |
| Dilated cardiomyopathy(K) | | 4.10E-07 | 1.48E-05 | *TPM3, TPM2, TPM1, MYL2, ATP2A2, TNNC1, MYH7, TTN, DES* |  |
| Ribosome(K) | | 1.71E-06 | 5.63E-05 | *RPL8, RPS14, RPS17, RPS11, RPS9, RPS5, RPLP1, RPLP0, RPS3A, RPS3* |  |
| Carbon metabolism(K) | | 2.87E-06 | 8.90E-05 | *ENO3, GOT2, ACO2, PGAM2, HADHA, PKM, ALDOA, GAPDH, MDH2* |  |
| Focal adhesion(K) | | 1.03E-05 | 2.97E-04 | *MYLK2, ACTN3, ACTN2, COL4A1, MYL2, RAF1, MYL12A, MYLPF, COL1A1, COL1A2, COL3A1* |  |
| Biosynthesis of amino acids(K) | | 1.28E-05 | 3.47E-04 | *ENO3, GOT2, ACO2, PGAM2, PKM, ALDOA, GAPDH* |  |
| Adrenergic signaling in cardiomyocytes(K) | | 2.42E-05 | 6.06E-04 | *TPM3, TPM2, TPM1, MYL2, ATF4, ATP2A2, TNNC1, MYH7, ATP1A2* |  |
| Glycolysis / Gluconeogenesis(K) | | 7.37E-05 | 1.77E-03 | *ENO3, LDHA, PGAM2, PKM, ALDOA, GAPDH* |  |
| Mitophagy(R) | | 8.65E-05 | 1.82E-03 | *VDAC1, SQSTM1, UBB, UBC* |  |
| Nicotinic acetylcholine receptor signaling pathway(P) | | 8.65E-05 | 1.82E-03 | *MYH7B, ACTA1, MYH2, MYH7* |  |
| Tight junction(K) | | 9.68E-05 | 1.94E-03 | *ACTN3, ACTN2, MYL2, MYL12A, MYH7B, MYLPF, MYH2, MYH7* |  |
| Amoebiasis(K) | | 1.36E-04 | 2.59E-03 | *ACTN3, ACTN2, COL4A1, COL1A1, COL1A2, HSPB1, COL3A1* |  |
| Metabolism of carbohydrates(R) | | 1.88E-04 | 3.57E-03 | *ENO3, UBB, UBC, GOT2, PYGM, PGAM2, PKM, ALDOA, GAPDH, MDH2* |  |
| Regulation of mRNA stability by proteins that bind AU-rich elements(R) | | 2.82E-04 | 5.07E-03 | *PSMD2, UBB, UBC, HSPA8, EIF4G1, HSPB1* |  |
| cGMP-PKG signaling pathway(K) | | 3.49E-04 | 5.93E-03 | *MYLK2, VDAC1, RAF1, ATF4, ATP2A2, SLC25A4, MYH7, ATP1A2* |  |
| Glucagon signaling pathway(K) | | 6.53E-04 | 0.0105 | *ATF4, ACACB, LDHA, PYGM, PGAM2, PKM* |  |
| Parkinson's disease(K) | | 6.97E-04 | 0.0112 | *VDAC1, UQCRC2, ATP5B, UBB, COX4I1, SLC25A4, ATP5A1* |  |
| Pyruvate metabolism(K) | | 8.21E-04 | 0.0123 | *ACACB, LDHA, PKM, MDH2* |  |
| downregulated of mta-3 in er-negative breast tumors(B) | | 8.97E-04 | 0.0135 | *TUBA4A, HSPB1, ALDOA* |  |
| Integrin signalling pathway(P) | | 1.29E-03 | 0.018 | *ACTN3, ACTN2, COL4A1, RAF1, COL1A1, COL1A2, COL3A1* |  |
| Cellular response to heat stress(R) | | 1.33E-03 | 0.0187 | *VCP, HSPA8, HSP90AB1, HSP90AA1, CRYAB* |  |
| Nephrin interactions(R) | | 1.59E-03 | 0.0207 | *ACTN3, ACTN2, SPTBN1* |  |
| Regulation of actin cytoskeleton(K) | | 1.71E-03 | 0.021 | *MYLK2, ACTN3, ACTN2, MYL2, RAF1, MYL12A, MYLPF, GSN* |  |
| Response to elevated platelet cytosolic Ca2+(R) | | 1.75E-03 | 0.021 | *SPARC, ACTN2, TUBA4A, ALDOA, TTN* |  |
| Protein processing in endoplasmic reticulum(K) | | 1.88E-03 | 0.0226 | *ATF4, VCP, HSPA8, HSP90AB1, HSP90AA1, RAD23B, CRYAB* |  |
| Ion channel transport(R) | | 2.08E-03 | 0.025 | *RAF1, UBB, UBC, RYR1, ATP2A2, TRDN, ATP1A2* |  |
| Protein digestion and absorption(K) | | 2.48E-03 | 0.0272 | *COL4A1, COL1A1, COL1A2, ATP1A2, COL3A1* |  |
| Calcium signaling pathway(K) | | 2.59E-03 | 0.0285 | *MYLK2, VDAC1, RYR1, ATP2A2, TNNC1, TNNC2, SLC25A4* |  |
| Signaling by Type 1 Insulin-like Growth Factor 1 Receptor (IGF1R)(R) | | 3.53E-03 | 0.0371 | *PSMD2, ACTN2, RAF1, UBB, UBC, SPTBN1, EIF4G1, EIF4B* |  |
| Estrogen signaling pathway(K) | | 3.71E-03 | 0.0371 | *RAF1, ATF4, HSPA8, HSP90AB1, HSP90AA1* |  |
| Hedgehog ligand biogenesis(R) | | 3.99E-03 | 0.0399 | *PSMD2, VCP, UBB, UBC* |  |
| Propanoate metabolism(K) | | 4.54E-03 | 0.0454 | *ACACB, LDHA, HADHA* |  |
| Beta1 integrin cell surface interactions(N) | | 4.97E-03 | 0.0471 | *COL4A1, COL1A1, COL1A2, COL3A1* |  |
| Central carbon metabolism in cancer(K) | | 5.24E-03 | 0.0471 | *RAF1, LDHA, PGAM2, PKM* |  |
| ISG15 antiviral mechanism(R) | | 5.24E-03 | 0.0471 | *UBB, UBC, EIF4G2, EIF4G1* |  |
| **XISQUETA** | | | | |  |
|  |  |  |  |  |  |
| **GeneSet** | | ***P*-value** | **FDR** | **Nodes** |  |
| Striated Muscle Contraction(R) | | 1.11E-16 | 3.87E-14 | *ACTN3, TPM3, ACTN2, TPM2, TPM1, MYL1, MYL2, NEB, MYBPC1, MYBPC2, TNNC1, TNNC2, TNNT1, TTN, TNNI1, TNNI2, DES, TCAP, VIM* |  |
| The citric acid (TCA) cycle and respiratory electron transport(R) | | 3.11E-15 | 5.41E-13 | *MT-ND6, MT-ND5, MT-CO1, UQCRC1, UQCRC2, ATP5B, LDHA, CYC1, NDUFV1, ACO2, MT-CYB, COX4I1, MT-ATP6, IDH3B, UQCRFS1, NDUFS2, ATP5A1, COX5B, MDH2* |  |
| Cardiac muscle contraction(K) | | 8.37E-14 | 9.71E-12 | *TPM3, TPM2, TPM1, MYL2, UQCRC1, UQCRC2, CYC1, COX4I1, ATP2A2, UQCRFS1, TNNC1, MYH7, ATP1A2, COX5B* |  |
| Glycolysis / Gluconeogenesis(K) | | 1.11E-10 | 9.66E-09 | *ENO3, LDHA, PGK1, GPI, ALDH2, PGM1, TPI1, PGAM2, PKM, ALDOA, GAPDH* |  |
| Eukaryotic Translation Elongation(R) | | 1.67E-09 | 1.13E-07 | *RPL8, RPS14, RPS17, RPS11, RPS5, EEF2, RPL3L, RPLP1, RPLP0, EEF1G, RPS3* |  |
| Carbon metabolism(K) | | 2.03E-09 | 1.13E-07 | *ENO3, PGK1, GOT2, ACO2, GPI, IDH3B, TPI1, PGAM2, PKM, ALDOA, GAPDH, MDH2* |  |
| Parkinson's disease(K) | | 2.42E-09 | 1.13E-07 | *UQCRC1, VDAC1, UQCRC2, ATP5B, UBB, CYC1, NDUFV1, COX4I1, UQCRFS1, NDUFS2, SLC25A4, ATP5A1, COX5B* |  |
| Metabolism of carbohydrates(R) | | 2.64E-09 | 1.13E-07 | *ENO3, PHKA1, UBB, UBC, PGK1, GOT2, GPI, PYGM, GYS1, PGM1, TPI1, PGAM2, PKM, ALDOA, GAPDH, MDH2* |  |
| Biosynthesis of amino acids(K) | | 5.10E-09 | 1.94E-07 | *ENO3, PGK1, GOT2, ACO2, IDH3B, TPI1, PGAM2, PKM, ALDOA, GAPDH* |  |
| Alzheimer's disease(K) | | 1.74E-08 | 5.91E-07 | *UQCRC1, UQCRC2, ATP5B, CYC1, NDUFV1, COX4I1, ATP2A2, ATP2A1, UQCRFS1, NDUFS2, GAPDH, ATP5A1, COX5B* |  |
| Nonsense-Mediated Decay (NMD)(R) | | 1.42E-07 | 4.41E-06 | *RPL8, RPS14, RPS17, RPS11, RPS5, RPL3L, EIF4G1, RPLP1, RPLP0, RPS3* |  |
| Hypertrophic cardiomyopathy (HCM)(K) | | 1.95E-07 | 5.60E-06 | *TPM3, TPM2, TPM1, MYL2, ATP2A2, TNNC1, MYH7, TTN, DES* |  |
| Eukaryotic Translation Termination(R) | | 2.16E-07 | 5.60E-06 | *RPL8, RPS14, RPS17, RPS11, RPS5, RPL3L, RPLP1, RPLP0, RPS3* |  |
| Eukaryotic Translation Initiation(R) | | 2.75E-07 | 6.61E-06 | *RPL8, RPS14, RPS17, RPS11, RPS5, RPL3L, EIF4G1, RPLP1, RPLP0, RPS3* |  |
| Dilated cardiomyopathy(K) | | 3.48E-07 | 8.00E-06 | *TPM3, TPM2, TPM1, MYL2, ATP2A2, TNNC1, MYH7, TTN, DES* |  |
| Huntington's disease(K) | | 6.45E-07 | 1.35E-05 | *UQCRC1, VDAC1, UQCRC2, ATP5B, CYC1, NDUFV1, COX4I1, UQCRFS1, NDUFS2, SLC25A4, ATP5A1, COX5B* |  |
| Oxidative phosphorylation(K) | | 1.10E-06 | 2.20E-05 | *UQCRC1, UQCRC2, ATP5B, CYC1, NDUFV1, COX4I1, UQCRFS1, NDUFS2, ATP5A1, COX5B* |  |
| SRP-dependent cotranslational protein targeting to membrane(R) | | 1.35E-06 | 2.56E-05 | *RPL8, RPS14, RPS17, RPS11, RPS5, RPL3L, RPLP1, RPLP0, RPS3* |  |
| Selenoamino acid metabolism(R) | | 1.46E-06 | 2.62E-05 | *RPL8, RPS14, RPS17, RPS11, RPS5, RPL3L, RPLP1, RPLP0, RPS3* |  |
| Metabolic pathways(K) | | 8.89E-06 | 1.51E-04 | *ENO3, UQCRC1, UQCRC2, AK1, ATP5B, LDHA, PGK1, CYC1, NDUFV1, GOT2, ACO2, GPI, COX4I1, PYGM, ALDH2, IDH3B, UQCRFS1, PGM1, TPI1, PGAM2, PKM, NDUFS2, ALDOA, GAPDH, ATP5A1, COX5B, CKMT2, CKM, MDH2* |  |
| Ribosome(K) | | 1.13E-05 | 1.82E-04 | *RPL8, RPS14, RPS17, RPS11, RPS5, RPL3L, RPLP1, RPLP0, RPS3* |  |
| Adrenergic signaling in cardiomyocytes(K) | | 2.08E-05 | 3.11E-04 | *TPM3, TPM2, TPM1, MYL2, ATF4, ATP2A2, TNNC1, MYH7, ATP1A2* |  |
| Non-alcoholic fatty liver disease (NAFLD)(K) | | 2.43E-05 | 3.64E-04 | *UQCRC1, UQCRC2, ATF4, CYC1, NDUFV1, COX4I1, UQCRFS1, NDUFS2, COX5B* |  |
| Glucagon signaling pathway(K) | | 8.00E-05 | 1.04E-03 | *PHKA1, ATF4, LDHA, PYGM, GYS1, PGAM2, PKM* |  |
| Mitophagy(R) | | 8.03E-05 | 1.04E-03 | *VDAC1, SQSTM1, UBB, UBC* |  |
| Calcium signaling pathway(K) | | 8.89E-05 | 1.16E-03 | *MYLK2, PHKA1, VDAC1, RYR1, ATP2A2, ATP2A1, TNNC1, TNNC2, SLC25A4* |  |
| cGMP-PKG signaling pathway(K) | | 3.06E-04 | 3.68E-03 | *MYLK2, VDAC1, ATF4, ATP2A2, ATP2A1, SLC25A4, MYH7, ATP1A2* |  |
| Smooth Muscle Contraction(R) | | 3.74E-04 | 4.48E-03 | *TPM3, TPM2, TPM1, MYLPF* |  |
| HIF-1-alpha transcription factor network(N) | | 5.84E-04 | 7.00E-03 | *LDHA, PGK1, PGM1, PKM, ALDOA* |  |
| 2-Oxocarboxylic acid metabolism(K) | | 7.20E-04 | 7.92E-03 | *GOT2, ACO2, IDH3B* |  |
| Pyruvate metabolism(K) | | 7.64E-04 | 8.40E-03 | *LDHA, ALDH2, PKM, MDH2* |  |
| Antigen processing and presentation(K) | | 1.16E-03 | 0.0116 | *B2M, HLA-E, HSPA8, HSP90AB1, HSP90AA1* |  |
| Cellular response to heat stress(R) | | 1.22E-03 | 0.0122 | *VCP, HSPA8, HSP90AB1, HSP90AA1, CRYAB* |  |
| Nicotinic acetylcholine receptor signaling pathway(P) | | 1.51E-03 | 0.0144 | *ACTA1, MYH2, MYH7* |  |
| Response to elevated platelet cytosolic Ca2+(R) | | 1.61E-03 | 0.0144 | *SPARC, ACTN2, TUBA4A, ALDOA, TTN* |  |
| Arginine and proline metabolism(K) | | 1.73E-03 | 0.0155 | *GOT2, ALDH2, CKMT2, CKM* |  |
| Ion channel transport(R) | | 1.86E-03 | 0.0168 | *SLN, UBB, UBC, RYR1, ATP2A2, ATP2A1, ATP1A2* |  |
| Starch and sucrose metabolism(K) | | 2.60E-03 | 0.0228 | *GPI, PYGM, GYS1, PGM1* |  |
| Tight junction(K) | | 2.85E-03 | 0.0228 | *ACTN3, ACTN2, MYL2, MYLPF, MYH2, MYH7* |  |
| Pentose phosphate pathway(K) | | 3.28E-03 | 0.0262 | *GPI, PGM1, ALDOA* |  |
| Citrate cycle (TCA cycle)(K) | | 3.60E-03 | 0.0288 | *ACO2, IDH3B, MDH2* |  |
| HIF-1 signaling pathway(K) | | 4.04E-03 | 0.0323 | *ENO3, LDHA, PGK1, ALDOA, GAPDH* |  |
| Cardiac conduction(R) | | 4.73E-03 | 0.0359 | *SLN, RYR1, ATP2A2, ATP2A1, ATP1A2* |  |
| Focal adhesion(K) | | 5.13E-03 | 0.0359 | *MYLK2, ACTN3, ACTN2, COL4A1, MYL2, MYLPF, COL3A1* |  |
| Mitotic G2-G2/M phases(R) | | 5.51E-03 | 0.0386 | *TUBA4A, UBB, UBC, HSP90AA1, DYNC1H1* |  |
| Arrhythmogenic right ventricular cardiomyopathy (ARVC)(K) | | 6.90E-03 | 0.0483 | *ACTN3, ACTN2, ATP2A2, DES* |  |

**Supplementary Table S5. Missense and nonsense mutations detected in at least two breed pools and mapping to genes involved in the determination of meat quality traits.**

| **Gene name** | **Location** | **Allele** | **Consequence** | **SIFT** | **Existing**  **variation** | **SNPs found in:** | | | | |
| --- | --- | --- | --- | --- | --- | --- | --- | --- | --- | --- |
|  |  |  |  |  |  | **Xisqueta** | **Canaria de Pelo** | **Roja Mallorquina** | **Ripollesa** | **Gallega** |
| Calpain 1 (*CAPN1*) | 21:42713857-42713857 | C | missense variant, splice region variant | - | - |  |  | ✓ | ✓ |  |
| Calpain 3 (*CAPN3*) | 7:34747195-34747195 | T | missense variant | tolerated low confidence (0.19) | rs429515616 |  | ✓ | ✓ | ✓ | ✓ |
| Calpastatin (*CAST*) | 5:93369797-93369797 | G | missense variant | tolerated (1) | rs162299347 | ✓ | ✓ | ✓ | ✓ | ✓ |
| Calpastatin (*CAST*) | 5:93457491-93457491 | A | missense variant | tolerated (0.4) | - |  |  | ✓ | ✓ |  |
| Carboxypeptidase E (*CPE*) | 17:447499-447499 | A | missense variant | tolerated (1) | - | ✓ | ✓ | ✓ | ✓ |  |
| Cathepsin B (*CTSB*) | 2:104431049-104431049 | T | missense variant | - | rs160131646 |  |  | ✓ | ✓ | ✓ |
| Cathepsin B (*CTSB*) | 2:104431734-104431734 | G | missense variant | - | - |  | ✓ |  |  | ✓ |
| Fatty Acid Binding Protein 4 (*FABP4*) | 9:57537775-57537775 | G | missense variant | deleterious (0) | - | ✓ | ✓ |  |  |  |
| Growth Hormone Receptor (*GHR*) | 16:31833100-31833100 | G | missense variant | - | rs161146164 | ✓ |  | ✓ | ✓ | ✓ |
| Growth Hormone Receptor (*GHR*) | 16:31833271-31833271 | T | missense variant | - | rs55631463 | ✓ | ✓ | ✓ | ✓ | ✓ |
| Growth Hormone Receptor (*GHR*) | 16:31833547-31833547 | A | missense variant | - | rs413776054 | ✓ |  | ✓ | ✓ | ✓ |
| Growth Hormone Receptor (*GHR*) | 16:31859509-31859509 | T | missense variant | - | rs404237321 | ✓ |  | ✓ |  | ✓ |
| Myogenic Differentiation 1 (*MYOD1*) | 15:34370835-34370835 | T | missense variant | - | rs868996532 | ✓ | ✓ | ✓ |  | ✓ |
| Myogenic Differentiation 1 (*MYOD1*) | 15:34370841-34370841 | T | missense variant | - | rs868996533 | ✓ | ✓ | ✓ |  | ✓ |
| Myogenic Differentiation 1 (*MYOD1*) | 15:34370845-34370845 | T | missense variant | - | rs868996535 | ✓ | ✓ | ✓ |  | ✓ |
| Myogenic Differentiation 1 (*MYOD1*) | 15:34370848-34370848 | T | missense variant | - | rs868996537 | ✓ | ✓ | ✓ |  | ✓ |
| Myogenic Differentiation 1 (*MYOD1*) | 15:34370853-34370853 | C | missense variant | - | - | ✓ | ✓ | ✓ |  | ✓ |
| Myogenic Differentiation 1 (*MYOD1*) | 15:34370855-34370855 | T | missense variant | - | - | ✓ | ✓ | ✓ |  | ✓ |
| Myogenic Factor 5 (*MYF5*) | 3:116622317-116622317 | G | missense variant | deleterious (0.02) | rs412277497 | ✓ | ✓ |  | ✓ | ✓ |
| Myogenin (*MYOG*) | 12:197606-197606 | T | missense variant | tolerated (1) | rs596811725 |  | ✓ | ✓ |  |  |
| Phosphoglycerate Mutase 2 (*PGAM2*) | 19:296078-296078 | G | missense variant | deleterious (0.01) | rs193644571 | ✓ |  |  |  | ✓ |
| Phosphorylase Kinase Catalytic Subunit Gamma 1 (*PHKG1*) | 24:27732379-27732379 | G | missense variant | tolerated (0.05) | - |  |  | ✓ | ✓ |  |
| Protein Kinase AMP-Activated Non-Catalytic Subunit Gamma 3 (*PRKAG3*) | 2:219781521-219781521 | G | missense variant | tolerated (1) | rs161743984 | ✓ | ✓ | ✓ |  | ✓ |
| Protein Kinase AMP-Activated Non-Catalytic Subunit Gamma 3 (*PRKAG3*) | 2:219783988-219783988 | A | missense variant | deleterious (0) | rs593823419 | ✓ |  |  | ✓ |  |
| Protein Kinase AMP-Activated Non-Catalytic Subunit Gamma 3 (*PRKAG3*) | 2:219785416-219785416 | G | missense variant | tolerated (0.05) | - |  |  |  | ✓ | ✓ |
| Protein Kinase AMP-Activated Non-Catalytic Subunit Gamma 3 (*PRKAG3*) | 2:219787003-219787003 | A | missense variant | tolerated low onfidence (0.11) | - | ✓ | ✓ | ✓ | ✓ | ✓ |
| Protein Kinase AMP-Activated Non-Catalytic Subunit Gamma 3 (*PRKAG3*) | 2:219787010-219787010 | T | missense variant | deleterious low confidence (0) | - | ✓ | ✓ | ✓ | ✓ | ✓ |
| Ryanodine Receptor 1 (*RYR1*) | 14:47448569-47448569 | G | missense variant | - | - |  |  |  | ✓ | ✓ |
| Ryanodine Receptor 1 (*RYR1*) | 14:47448592-47448592 | T | missense variant | - | - | ✓ | ✓ | ✓ | ✓ | ✓ |
| Ryanodine Receptor 1 (*RYR1*) | 14:47466574-47466574 | T | missense variant | - | rs419780331 | ✓ | ✓ | ✓ |  | ✓ |
| Ryanodine Receptor 1 (*RYR1*) | 14:47466972-47466972 | A | missense variant | - | - | ✓ | ✓ | ✓ | ✓ | ✓ |
| Ryanodine Receptor 1 (*RYR1*) | 14:47470077-47470077 | C | missense variant | - | rs401203710 | ✓ | ✓ | ✓ | ✓ | ✓ |
| Ryanodine Receptor 1 (*RYR1*) | 14:47473856-47473856 | T | missense variant | - | rs415434417 | ✓ | ✓ | ✓ | ✓ | ✓ |
| Ryanodine Receptor 1 (*RYR1*) | 14:47473863-47473863 | G | missense variant | - | - | ✓ | ✓ | ✓ | ✓ | ✓ |
| Ryanodine Receptor 1 (*RYR1*) | 14:47473904-47473904 | G | missense variant | - | rs428451012 |  | ✓ | ✓ | ✓ |  |
| Ryanodine Receptor 1 (*RYR1*) | 14:47481616-47481616 | C | missense variant | - | - | ✓ | ✓ |  | ✓ | ✓ |
| Ryanodine Receptor 1 (*RYR1*) | 14:47493917-47493917 | C | missense variant | - | - |  |  | ✓ | ✓ | ✓ |
| Ryanodine Receptor 1 (*RYR1*) | 14:47495209-47495209 | C | missense variant | - | - | ✓ | ✓ | ✓ | ✓ | ✓ |
| Ryanodine Receptor 1 (*RYR1*) | 14:47535584-47535584 | C | missense variant | - | - |  |  | ✓ | ✓ |  |
| Troponin C2 (*TNNC2*) | 13:74137159-74137159 | A | missense variant, splice region variant | tolerated (1) | - | ✓ | ✓ | ✓ | ✓ |  |

**Supplementary** **Table S6. List of genes with known associations with meat quality traits.**

| **Gene** | **Reference** |
| --- | --- |
| Calpain 1 (*CAPN1*) | Lambe NR, Krzecio-Nieczyporuk E,Kocwin-Podsiadła M, Bünger L. Influence of Major Genes on Meat Quality. In: Przybylski W, Hopkins D, Editors. Meat Quality, Genetic and Environmental Factors. Boca Raton: CRC Press; 2015, p. 287–332. |
| Calpain 3 (*CAPN3*) | Warner RD, Greenwood PL, Pethick DW, Ferguson DM. Genetic and environmental effects on meat quality. Meat Sci. 2010;86:171-83. |
| Calpastatin (*CAST*) | Lambe NR, Krzecio-Nieczyporuk E,Kocwin-Podsiadła M, Bünger L. Influence of Major Genes on Meat Quality. In: Przybylski W, Hopkins D, Editors. Meat Quality, Genetic and Environmental Factors. Boca Raton: CRC Press; 2015, p. 287–332. |
| Carboxypeptidase E (*CPE*) | Ribeca C, Bittante G, Albera A, Bonfatti V, Maretto F, Gallo L. Investigation on variability of candidate genes for meat quality traits in Piemontese cattle. Ital J Anim Sci. 2009;8:132-140 |
| Cathepsin B (*CTSB*) | Ribeca C, Bittante G, Albera A, Bonfatti V, Maretto F, Gallo L. Investigation on variability of candidate genes for meat quality traits in Piemontese cattle. Ital J Anim Sci. 2009;8:132-138 |
| Fatty Acid Binding Protein 4 (*FABP4*) | Lambe NR, Krzecio-Nieczyporuk E,Kocwin-Podsiadła M, Bünger L. Influence of Major Genes on Meat Quality. In: Przybylski W, Hopkins D, editors. Meat Quality, Genetic and Environmental Factors. Boca Raton: CRC Press; 2015, p. 287–332. |
| Growth Hormone Receptor (*GHR*) | Ribeca C, Bittante G, Albera A, Bonfatti V, Maretto F, Gallo L. Investigation on variability of candidate genes for meat quality traits in Piemontese cattle. Ital J Anim Sci. 2009;8:132-135 |
| Myogenic Factor 5 (*MYF5*) | Guo Y, Li J, Shang J, Jin Y. Development of Muscle-related Genes and Their Effects on Meat Quality. Energy Procedia. 2012;16:229–233. |
| Myogenic Differentiation 1 (*MYOD1*) | Guo Y, Li J, Shang J, Jin Y. Development of Muscle-related Genes and Their Effects on Meat Quality. Energy Procedia. 2012;16:229–233. |
| Myogenin (*MYOG*) | Lambe NR, Krzecio-Nieczyporuk E,Kocwin-Podsiadła M, Bünger L. Influence of Major Genes on Meat Quality. In: Przybylski W, Hopkins D, Editors. Meat Quality, Genetic and Environmental Factors. Boca Raton: CRC Press; 2015, p. 287–332. |
| Myogenin (*MYOG*) | Guo Y, Li J, Shang J, Jin Y. Development of Muscle-related Genes and Their Effects on Meat Quality. Energy Procedia. 2012;16:229–233. |
| Phosphoglycerate Mutase 2 (*PGAM2*) | Fontanesi L, Davoli R, Nanni Costa L, Beretti F, Scotti E, Tazzoli M, et al. Investigation of candidate genes for glycolytic potential of porcine skeletal muscle: Association with meat quality and production traits in Italian Large White pigs. Meat Sci. 2008;80:780-7 |
| Phosphorylase Kinase Catalytic Subunit Gamma 1 (*PHKG1*) | Ma J, Yang J, Zhou L, Ren J, Liu X, Zhang H, et al. A splice mutation in the PHKG1 gene causes high glycogen content and low meat quality in pig skeletal muscle. PLOS Genet. 2014;10:e1004710. |
| Protein Kinase AMP-Activated Non-Catalytic Subunit Gamma 3 (PRKAG3) | Milan D, Jeon JT, Looft C, et al.2000. A mutation in PRKAG3 associated with excess glycogen content in pig skeletal muscle. Science 288:1248-51. |
| Ryanodine Receptor 1 (*RYR1*) | Lambe NR, Krzecio-Nieczyporuk E,Kocwin-Podsiadła M, Bünger L. Influence of Major Genes on Meat Quality. In: Przybylski W, Hopkins D, Editors. Meat Quality, Genetic and Environmental Factors. Boca Raton: CRC Press; 2015, p. 287–332. |
| Troponin C2 (*TNNC2*) | Knapik J, Ropka-Molik K, Pieszka P. Genetic and nutritional factors determining the production and quality of sheep meat - a review. Ann Anim Sci. 2017;17:23–40. |
